# Small NRPS-like enzymes in *Aspergillus* sections *Flavi* and *Circumdati* selectively form substituted pyrazinone metabolites

**DOI:** 10.3389/ffunb.2022.1029195

**Published:** 2022-10-26

**Authors:** Matthew D. Lebar, Brian M. Mack, Carol H. Carter-Wientjes, Qijian Wei, Christopher P. Mattison, Jeffrey W. Cary

**Affiliations:** ^1^ Food and Feed Safety Research, Southern Regional Research Center, Agricultural Research Service, United States Department of Agriculture (USDA), New Orleans, LA, United States; ^2^ Food Processing and Sensory Quality Research, Southern Regional Research Center, Agricultural Research Service, United States Department of Agriculture (USDA), New Orleans, LA, United States

**Keywords:** mycotoxin, aspergillic acid, ATR, biosynthesis, aflatoxin, ochratoxin, flavacol

## Abstract

*Aspergillus* fungi produce mycotoxins that are detrimental to human and animal health. Two sections of aspergilli are of particular importance to cereal food crops such as corn and barley. *Aspergillus* section *Flavi* species like *A. flavus* and *A. parasiticus* produce aflatoxins, while section *Circumdati* species like *A. ochraceus* and *A. sclerotiorum* produce ochratoxin A. Mitigating these toxins in food and feed is a critical and ongoing worldwide effort. We have previously investigated biosynthetic gene clusters in *Aspergillus flavus* that are linked to fungal virulence in corn. We found that one such cluster, *asa*, is responsible for the production of aspergillic acid, an iron-binding, hydroxamic acid-containing pyrazinone metabolite. Furthermore, we found that the *asa* gene cluster is present in many other aflatoxin- and ochratoxin-producing aspergilli. The core gene in the *asa* cluster encodes the small nonribosomal peptide synthetase-like (NRPS-like) protein AsaC. We have swapped the *asaC* ortholog from *A. sclerotiorum* into *A. flavus*, replacing its native copy, and have also cloned both *asaC* orthologs into *Saccharomyces cerevisiae*. We show that AsaC orthologs in section *Flavi* and section *Circumdati*, while only containing adenylation-thiolation-reductase (ATR) domains, can selectively biosynthesize distinct pyrazinone natural products: deoxyaspergillic acid and flavacol, respectively. Because pyrazinone natural products and the gene clusters responsible for their production are implicated in a variety of important microbe-host interactions, uncovering the function and selectivity of the enzymes involved could lead to strategies that ultimately benefit human health.

## Introduction

*Aspergillus* fungi infect crops and can contaminate food and feed with mycotoxins (Taniwaki et al., 2018). *A. flavus* and many other section *Flavi* species produce aflatoxins, potent carcinogens, that are regulated in the food supply worldwide (Frisvad et al., 2019). Aflatoxins are hepatotoxic and can cause acute illness as well as liver cancer with prolonged exposure (Wogan, 1966; Groopman et al., 1988). *Aspergillus* section *Circumdati* species, such as *A. ochraceus*, produce ochratoxin A, a nephrotoxin, mutagen, and potential carcinogen (Visagie et al., 2014). Ochratoxin A is found in many food and feed products, particularly cereals and grapes, and can even contaminate processed meat if the animal consumed infected grain. *Aspergillus* infection of crops, and subsequent mycotoxin contamination of the food supply, can occur preharvest or postharvest (Agriopoulou et al., 2020). Identifying key plant-pathogen interactions could lead to mitigating preharvest infection by toxin-producing fungi. In addition to known toxins, *Aspergillus* species also have the capacity to produce many other secondary metabolites, some of which may be involved in infection (Kjaerbolling et al., 2018; Romsdahl and Wang, 2019). For example, cyclopiazonic acid has been identified as an *A. flavus* pathogenicity factor during corn infection that results in ear rot and subsequent aflatoxin contamination (Chalivendra et al., 2017). The biosynthetic gene clusters responsible for secondary metabolite production are abundant in *Aspergillus* fungi, however a majority of these clusters are uncharacterized (Uka et al., 2020; Robey et al., 2021). Characterizing these clusters and determining the function of their metabolite products could lead to preharvest strategies that target fungal metabolites essential to the infection process (Bignell et al., 2016).

**Figure 1:**
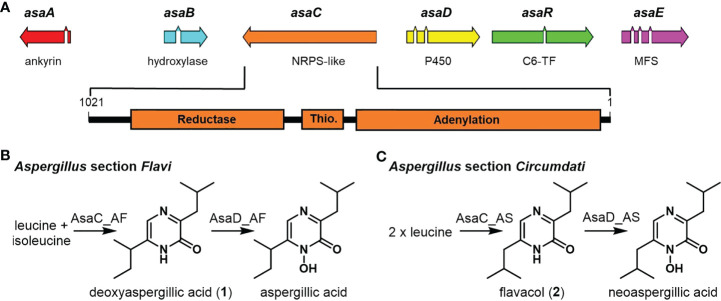
The pyrazinone precursors to aspergillic acids are formed by the NRPS-like enzyme AsaC in *Aspergillus* spp. **(A)** Schematic of the *asa* biosynthetic gene cluster responsible for aspergillic acid production contains the NRPS-like core gene *asaC*, which is comprised of Adenylation-Thiolation-Reductase (ATR) domains. Introns are shown as gaps in the genes. **(B)** Species in *Aspergillus* section *Flavi* such as *A. flavus* produce aspergillic acid from leucine and isoleucine through a deoxyaspergillic acid (**1**) intermediate. **(C)** Species in *Aspergillus* section *Circumdati* such as *A. sclerotiorum* produce neoaspergillic acid from two leucine residues from a flavacol (**2**) intermediate.

We have previously characterized the biosynthetic gene cluster *asa* in *A. flavus* and found it responsible for aspergillic acid production (Lebar et al., 2018). Aspergillic acid is a hydroxamic acid containing pyrazinone formed from leucine and isoleucine precursors that binds iron to form an Fe(III) trimer complex called ferriaspergillin (MacDonald, 1961; Pitt et al., 1983). The ability to produce aspergillic acid has been linked to fungal virulence in a corn kernel assay using *asa* knock out and genetically complimented strains (Lebar et al., 2018). The *asa* cluster in *A. flavus* encodes several biosynthetic proteins: a nonribosomal peptide synthetase-like (NRPS-like) enzyme (AsaC), a CYP450 oxidoreductase (AsaD), and a hydroxylase (AsaB), as well as an MFS transporter (AsaE), an Ankyrin domain protein (AsaA), and a zinc finger transcription factor (AsaR) (**Figure 1A**). We also previously reported that *asa* cluster orthologs were present in many aflatoxin-producing species (*Aspergillus* section *Flavi*) as well as ochratoxin-producing species (*Aspergillus* section *Circumdati*) (Lebar et al., 2019). The section *Circumdati* strains do not produce aspergillic acid but a similar analog named neoaspergillic acid, which is formed from two leucine residues (Micetich and MacDonald, 1965).

The core NRPS-like enzyme (AsaC), comprised of only Adenylation-Thiolation-Reductase (ATR) domains, is thought to be responsible for pyrazinone formation utilizing two amino acid residues. NRPS reductase domains liberate the NRP from the enzyme forming an aldehyde product (Forseth et al., 2013; Mullowney et al., 2018). AsaC is a noncanonical NRPS (“NRPS-like”) as it lacks a condensation domain (C), which is the domain that typically forms peptide bonds in NRPs. The pyrazinone precursors to aspergillic acid and neoaspergillic acid differ only by the location of one methyl group on one of the pendant aliphatic side chains (*sec*-butyl and isobutyl, respectively) (**Figures 1B** **, C**). Because the amino acid progenitors of these side chains are likely selected by the NRPS-like protein, we focused this study on determining the products of AsaC orthologs in both *A. flavus* and *A. sclerotiorum*. Investigating the selectivity of these small, very similar, noncanonical NRPSs would demonstrate their unique activity and could lead to bioengineering strategies to easily generate differently substituted pyrazinones and their iron-binding, hydroxamic acid analogs.

## Materials and methods

### Genomic analysis

The *A. flavus* 3357 *asa* biosynthetic gene cluster sequence (AFLA_023000 – AFLA_023050: *asaA*, *asaB*, *asaC*, *asaD*, *asaR*, and *asaE*, respectively) (Nierman et al., 2015; Lebar et al., 2018) was identified in other genomes using TBLASTN of the six proteins against all fungal genomes at JGI and all Pezizomycotina genomes from NCBI. The best hit for each protein was merged with the other best hits if they were within 2kb from each other, and then filtered for a total length of at least 10kb using a custom R script. The “annotate from” tool in Geneious Prime (version 2021.2) was used to annotate the six *asa* genes in each genome from their encoded proteins. The nonribosomal codes (A1-A10) of the AsaC orthologs were identified by aligning the GrsA-PheA to the *Aspergillus* spp. protein sequences using Muscle (Edgar, 2004). The phylogenetic tree of AsaC amino acid sequences was created with IQ-TREE (Nguyen et al., 2015) and the LG substitution matrix (Le and Gascuel, 2008) using the ultrafast bootstrapping approximation option. Geneious Prime was used to generate the identity matrix for the nucleotide and protein alignments.

### Strains and culture conditions

All strains used in this study are described in **Table 1**. A. *flavus* AF70 (SRRC 1529) and *A. sclerotiorum* (SRRC 2162 = NRRL 415 = CBS 549.65) wild-type strains are housed in the Southern Regional Research Center (SRRC) permanent culture collection in New Orleans, USA. *A. flavus* CA14 Δ*ku70*, *niaD*, Δ*pyrG*, *ptrA^S^* (SRRC 1709, referred to herein as CA14) was used as host for transformation and is sensitive to pyrithiamine (ptrA^S^) (Chang et al., 2010). The control strain was CA14 transformed with vector pPTRI (Takara Bio Inc., Shiga, Japan) that contains the pyrithiamine (*ptrA*) resistance gene (Chang et al., 2010). The *A. flavus* CA14 *asaD* (AFLA_023030) gene knock out mutant was generated from CA14 using pyrithiamine selection as described previously (Lebar et al., 2018). Fungal spore stocks were generated from cultures point inoculated on double strength V8 agar (50 mL V8 juice, 40 g agar, pH 5.2 per liter of medium) supplemented with 3.0 g ammonium sulfate and 1 mg/ml uracil (2X V8 ASU) and incubated at 30°C in the light (Chang et al., 1993). Generation of the *S. cerevisiae* strain expressing the *A. flavus asaC* NRPS-like gene (AFLA_023020, herein called asaC_AF) was described previously (Lebar et al., 2018). All chemicals were purchased from Sigma-Aldrich (St. Louis, MO, USA) unless otherwise noted.

**Table 1:** Strains used in the study.

Strain	Description	Reference
*Aspergillus flavus* AF70	SRRC 1529, isolated from soil in Arizona, USA	(Cotty, 1989)
*Aspergillus sclerotiorum*	SRRC 2162 = NRRL 415 = CBS 549.65, type strain, isolated from apple in Oregon, USA	(Visagie et al., 2014)
*Aspergillus flavus* CA14	SRRC 1709, Δ*ku70*, *niaD*, Δ*pyrG*, *ptrA^S^*	(Chang et al., 2010)
*Aspergillus flavus* CA14 Δ*asaD*	AFLA_023030 (CYP450) knockout mutant	(Lebar et al., 2018)
*Saccharomyces cerevisiae-asaC_AF*	BJ5464-NpgA expressing AFLA_023020 (NRPS-like) from *A. flavus*	(Lebar et al., 2018)
*A. flavus* CA14-*asaC_AF to asaC_AS*	AFLA_023020 ortholog from *A. sclerotiorum* swap mutant	this study
*Saccharomyces cerevisiae-asaC_AS*	BJ5464-NpgA expressing AFLA_023020 ortholog from *A. sclerotiorum*	this study

### Vector construction and fungal transformation of the CA14 *asaC_AF* to *asaC_AS* swap mutant

The *A. flavus* CA14 *asaC_AF to asaC_AS* gene swap mutant was generated from CA14 using *pyrG* selection. *A. flavus* CA14 (PT^S^) was transformed using an *asaC_AS* vector constructed by NEBuilder HiFi DNA Assembly method according to the manufacturer’s protocol (NEB E2621S, Ipswich, MA, USA). Briefly, the full length NRPS-like gene from *A. sclerotiorum* (*asaC_AS*) as well as the *asaC_AF* NRPS-like gene’s (AFLA_023020) native promotor and terminator were amplified by PCR using Q5 High-Fidelity DNA Polymerase (NEB M0492S). *A. sclerotiorum* gDNA was the template for amplification of the NRPS of *asaC_AS* using the primer pair Asc_NRPS_F and Asc_NRPS_R (**Table 2**). *A. flavus* 3357 gDNA served as the template for amplification of the native *asaC_AF* promotor and terminator regions using two primer pairs, AF_NRPS_prom_F/prom_R and AF_NRPS_Term_F/Term_R (**Table 2**). The three amplified PCR fragments were then assembled and cloned into a linearized pUC_pyrG plasmid (digest of *Not*I and *Bam*HI) with the *pyrG* selection marker (**Figure S1**). PCR using the primer pair Asc_NRPS_RP nest_F/nest_R (**Table 2**) was used to screen and confirm the correct clone (**Figure S2**) as well as *Bam*HI restriction enzyme digestion (**Figure S3**). Recombinant plasmids were linearized with *Not*I and used to transform protoplasts of *A. flavus* CA14 that were generated as described in Cary et al. (2006). Conidia were inoculated in Czapek-Dox (CZ; BD Difco, Franklin Lakes, NJ, USA) broth supplemented with 10 mM ammonium sulfate and 1 mg/ml uracil (CZ-ASU). Transformants were regenerated on CZ-AS without uracil agar plates. Correct transformants were confirmed by PCRs using the primer pair Asc_NRPS_F1 and Asc_NRPS_R1 (**Table 2**; **Figure S4**) and Asc_NRPS_F12 and pyrG-R (300) (**Table 2**; **Figure S5**).

**Table 2:** Oligonucleotide primers used in the study.

Primer name	Sequence
AF_NRPS_prom_F	gtgaattcgagctcggtacccgggcggccgCTTTGCCACTGTTCACTCCTGG
AF_NRPS_prom_R	aggagagattggggaatccagacatTGTGAGTGTGGTTTGTCAGGGAG
Asc_NRPS_F	tgctccctgacaaaccacactcacaATGTCTGGATTCCCCAATCTC
Asc_NRPS_R	ctaccatgtatgccatctagaagtaTTACACTCTAGAAGGGACAAG
AF_NRPS_Term_F	gggccttgtcccttctagagtgtaaTACTTCTAGATGGCATACATGGTAG
AF_NRPS_Term_R	tggtatattgttctgagatccataggatCCGCTAGAAGAACGCCTCATAC
Asc_NRPS_RP nest_F	GGAATGTTCCTCCCGGTCTC
Asc_NRPS_RP nest_R	CTGACGACAGGGATCAGGTG
Asc_NRPS_F1	TTTGCCACTGTTCACTCCTG
Asc_NRPS_R1	GGGGAATCCAGACATTGTGAGT
Asc_NRPS_F12	AGGCGCTGTCGCAGGATAAG
pyrG-R (300)	Atctggaccaaacacatcgat
Asc_023020_F	gattataaggatgatgatgataagactagtATGTCTGGATTCCCCAATCTC
Asc_023020_R	atcggtccgcacaaatttgtcatttaaaTTACACTCTAGAAGGGACAAG

### Yeast expression system

*Saccharomyces cerevisiae* strain BJ5464-NpgA (MATα ura3-52 his3-Δ200 leu2-Δ1 trp1 pep4::HIS3 prb1Δ1.6R can1 GAL) was used as the yeast expression host (Mootz et al., 2002; Ma et al., 2019). The full length *asaC* NRPS-like gene (AFLA_023020) homolog from *A. sclerotiorum* (*asaC_AS*) was PCR amplified from *A. sclerotiorum* genomic DNA according to the manufacturer’s protocol using Q5 High-Fidelity DNA Polymerase (NEB M0492S) and the primer pair Asc_023020_F and Asc_023020_R (**Table 2**; **Figure S6**). The *asaC_AS* PCR product was subsequently cloned into the yeast 2 μm expression plasmid (a kind gift from Yi Tang, University of California, Los Angeles) XW55 (SpeI-SwaI digested) using the In-Fusion HD Cloning Kit (Takara Bio, Mountain View, CA, USA) to generate plasmid XW55-asaC_AS (**Figure S7**). The yeast expression plasmid was used to transform Stellar competent *E. coli* cells (Takara Bio) according to the manufacturer’s protocol. Cells were spread onto LB plates containing ampicillin (100μg/ml) and incubated at 37°C overnight. XW55-asaC_AS plasmid DNA was isolated from transformants using the Zyp-py™ Plasmid Miniprep Kit (ZymoResearch, Irvine, CA, USA). The yeast expression vectors were sequenced to confirm the correctness of the cloned genes. Two vacuolar proteases PEP4 and PRB1 were inactivated in the BJ5464-NpgA yeast host, which is critical to minimize proteolysis of the large recombinant proteins. The XW55-asaC_AS expression plasmid was transformed into *S. cerevisiae* BJ5464-NpgA by using S. c. EasyComp™ Transformation Kit (Thermo Fisher Scientific, Waltham, MA, USA). The presence of the XW55-asaC_AS plasmid DNA in transformed yeast was confirmed by KpnI and BamHI restriction enzyme digestion of isolated plasmid DNA (**Figure S8**) and by sequencing of the 3063 bp *asaC_AS* gene region amplified from isolated DNA using Q5 High-Fidelity DNA Polymerase. Yeast transformants harboring the XW55-asaC_AS vector were used for further biochemical analyses.

### Production, extraction, and analysis of hydroxamic acid trimer-iron(III) complexes

Ferriaspergillin and ferrineoaspergillin production by *A. flavus* (AF70, CA14 control, and the *asaC_AF* to *asaC_AS* swap mutant) and *A. sclerotiorum* strains was assessed by single point inoculation of spore suspensions on *Aspergillus flavus* and *parasiticus* agar (AFPA) medium [20 g yeast extract, 10 g bacto peptone, 0.5 g ferric ammonium citrate, 20 g agar, pH 5.0 per liter of medium, (Pitt et al., 1983)] incubated for 10 days at 30°C in the dark. The fungal colonies were excised from the medium. The fungus was lyophilized, pulverized, then extracted thrice (24 h, shaking at 200 RPM) with ethyl acetate containing 0.1% formic acid. The red-orange extract was filtered through filter paper (Whatman 1, GE Healthcare Life Sciences, USA) and concentrated *in vacuo*. The dried extracts were dissolved in methanol at 1 mg/ml and centrifuged to remove particulate before analysis on a Waters (Milford, MA, USA) ACQUITY UPLC system using PDA UV and QDa nominal mass detection as reported previously (Lebar et al., 2019). Separations were achieved with a BEH C18 1.7µm, 2.1 x 50 mm column and the following gradient solvent system: (0.5 ml/min, solvent A: 0.1% formic acid in water; solvent B: 0.1% formic acid in acetonitrile); 5% B (0-1.25 min), gradient to 25% B (1.25-1.5 min), gradient to 100% B (1.5-5.0 min), 100% B (5.0-7.5 min), then column equilibration 5% B (7.6-10.1 min). UV absorbance was recorded at λ = 200-500 nm and measured at λ = 310 nm.

### Production and extraction of substituted pyrazinones in Aspergilli and yeast

Conidia (10^6^/ml) of *A. flavus* CA14 Δ*asaD* (AFLA_023030) and *A. sclerotiorum* were used to inoculate 100 ml YE-glycerol broth [2% w/v yeast extract (BD, Sparks, MD), 1% v/v glycerol, pH = 6.5) (Woodward, 1947)]. Static cultures were incubated at 30°C for 14 days. After removal of the mycelial mat, the spent broth was adjusted to pH = 3.0 with 6 N hydrochloric acid then extracted with chloroform (100 ml). The organic layer was removed and concentrated on a rotary evaporator. A portion of the extract was redissolved in methanol at 100 µg/ml then centrifuged (20,000 × *g*, 2 min) to remove particulates for HRMS analysis. *Saccharomyces cerevisiae* strains transformed with *asaC_AF* and *asaC_AS* genes were grown in yeast extract-peptone-dextrose broth [1% yeast extract (BD, Sparks, MD), 2% peptone, 2% dextrose; YPD] as described previously (Cacho and Tang, 2016). YPD (5 ml) was inoculated with a single colony of *S. cerevisiae* BJ5464-NpgA grown on selective media and incubated at 28°C for 3 days. The cultures were centrifuged (20,000 × *g*, 2 min). The supernatant was transferred to a clean vial and extracted with ethyl acetate containing 0.1% formic acid (5 ml), concentrated *in vacuo*, and then dissolved in methanol (200 µl) for HRMS analysis.

### HRMS analysis of substituted pyrazinones

Analyses of deoxyaspergillic acid and flavacol in organic solvent extracts were conducted on a Waters ACQUITY UPLC system using photo diode array (PDA) UV and quadrupole time-of-flight (qTOF) mass detection with the following chromatographic conditions on a Waters BEH C18 1.7 μm, 2.1 mm × 50 mm column: 0.5 ml/min, solvent A (0.1% formic acid in water); solvent B (0.1% formic acid in acetonitrile); 25% B (0–1.5 min), gradient to 50% B (1.5–5.0 min), gradient to 100% B (5.0–5.1 min), 100% B (5.1–7.5 min), then column equilibration 5% B (7.6–10.0 min). High resolution mass (HRM) data were collected on a Waters Xevo G2-XS qTOF spectrometer equipped with a Z-spray ionization source running in ESI+ mode using Waters MassLynx 4.2 software. The qTOF conditions allowed for metabolite ionization (source temperature: 100°C; desolvation temperature: 250°C; desolvation gas flow: 600 L/h; cone gas flow: 50 L/h; capillary voltage: 3.0 kV; sampling cone voltage: 40 V). Analyses were performed in sensitivity and continuum mode, with a mass range of m/z 50–1200 and a scan time of 0.1 s. A data-independent acquisition method with elevated collision energy (MS^E^) was used with the following eV settings: 6 eV low energy and a high energy ramp from 10−45 eV. Mass calibration was performed with sodium formate. Lock mass data was acquired in MassLynx using leucine enkephalin as a reference at 20 sec. intervals with 3 scans to average. Data were analyzed on Waters UNIFI 1.9.4 software using the “accurate mass screening on MS^E^ data” analysis method with lock mass corrected by UNIFI.

### Protein modelling

The *Aspergillus* NRPS-like enzymes’ adenylation domains were modeled using the crystal structure of the *Brevibacillus brevis* gramicidin synthase-1 phenylalanine activation domain (PheA) complexed with cyclic-AMP and phenylalanine as a template (Conti et al., 1997). The PheA structure was obtained from the Protein Data Bank (pdb 1AMU) (Berman et al., 2000; Burley et al., 2017). The *A. flavus* and *A. sclerotiorum* adenylation domain models were generated using the homology modeler function within the Molecular Operating Environment (MOE 2020.0901, Chemical Computing Group, Montreal, QC, Canada) software. Structures were prepared using Protonate3D and QuickPrep within MOE to fill in missing atoms, build small loops, cap termini, form disulfide bonds, allow flipping of terminal amides, sulfonamide and imidazole groups, add hydrogens, and determine ionization state to optimize the hydrogen bond network. Substitution of isoleucine for leucine in the enzyme binding pocket was accomplished using the protein builder function in MOE and the orientation of the amino acid was optimized with QuickPrep in MOE as described above.

## Results

### The *asa* biosynthetic gene cluster is present in many *Aspergillus* section *Flavi* and section *Circumdati* species

**Figure 2:**
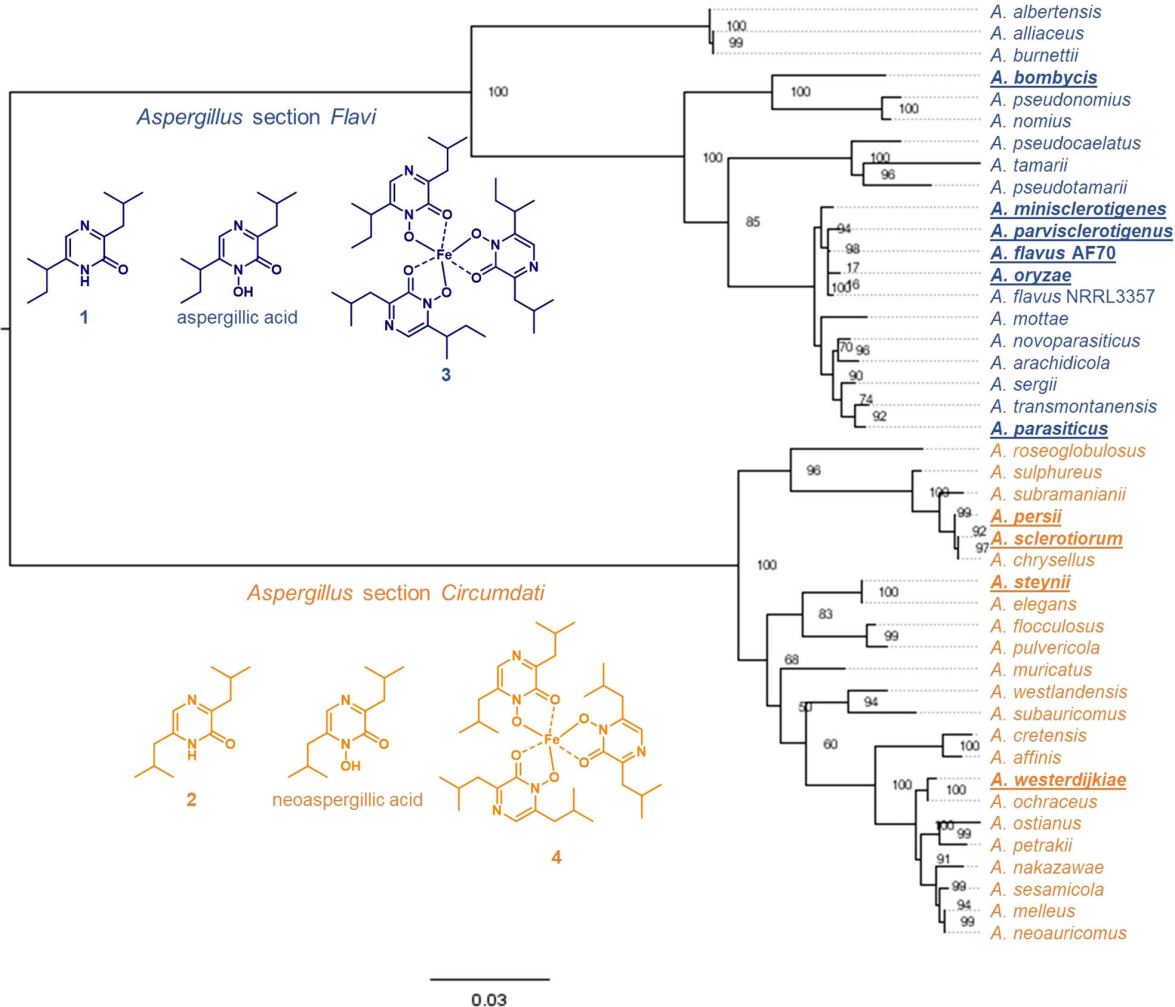
Sequence homology of AsaC in *Aspergillus* section *Flavi* is distinct from section *Circumdati* species. Phylogenetic tree of AsaC NRPS-like orthologs in *Aspergillus* section *Flavi* (blue) and *Circumdati* (orange) species. Chemical confirmation of the substituted pyrazinones and their derivatives produced by these species are in underlined bold font (Lebar et al., 2019). Blue: predominantly deoxyaspergillic acid-derived (**1**), including aspergillic acid and ferriaspergillin (**3**); orange: exclusively flavacol-derived (**2**), including neoaspergillic acid and ferrineoaspergillin (**4**). The node values represent the branch bootstrap values using the ultrafast bootstrap approximation.

TBLASTN queries of protein sequences in the *asa* cluster identified 19 *Aspergillus* section *Flavi* and 23 *Aspergillus* section *Circumdati* genomes that contained the entire cluster (**Figure 2**, **Table S1**). The identified clusters were quite similar. Although our query was designed to find the clustered genes in any order, all *asa* cluster orthologs that we found were ordered identically to *asa* in *A. flavus* 3357, the organism in which *asa* was first identified and characterized (Lebar et al., 2018). *Aspergillus* section *Flavi asa* cluster orthologs have mean pairwise nucleotide sequence identity of 79.0% to each other and 84.7% to *A. flavus* 3357 *asa*. *Aspergillus* section *Circumdati asa* cluster orthologs have a mean pairwise nucleotide sequence identity of 78.2% to each other and 50.7% to *A. flavus* 3357. The NRPS-like protein AsaC had similar homology between and within sections. **Figure 2** shows a phylogenetic tree comparing all AsaC orthologs encoded in *asa* clusters. *Aspergillus* section *Flavi* AsaC orthologs have a mean pairwise amino acid sequence identity of 93.1% to each other and 95.3% to *A. flavus* 3357 AsaC, while section *Circumdati* AsaC orthologs have a mean pairwise amino sequence identity of 94.6% to each other and 71.6% to *A. flavus* 3357.

### *A. flavus asaC_AS* swap mutant produces ferrineoaspergillin

**Figure 3:**
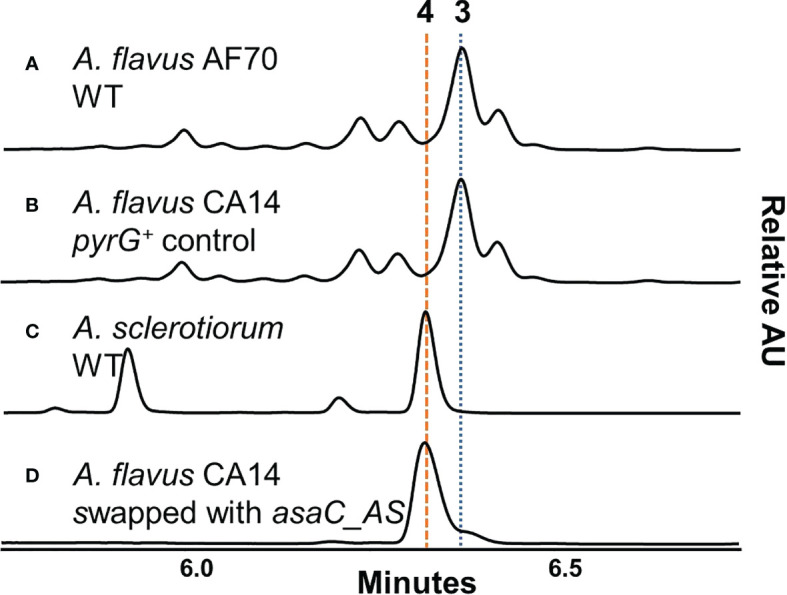
*A. flavus* containing *asaC* from *A. sclerotiorum* produces an iron(III)-bound trimer found naturally only in *Aspergillus* section *Circumdati* species. **(A–D)** UV chromatograms of *Aspergillus* extracts grown on AFPA measured in relative absorbance units (AU) at λ = 310 nm.

We have previously shown that many species from *Aspergillus* section *Flavi*, including *A. flavus* produce mainly ferriaspergillin (from aspergillic acid, See **Figure 2**, blue bolded font) and species from *Aspergillus* section *Circumdati*, including *A. sclerotiorum*, produce ferrineoaspergillin (**Figure 2**, orange bolded font) (Lebar et al., 2019). To further investigate the role of *asaC* in pyrazinone biosynthesis, the *asaC_AF* gene naturally occurring in *A. flavus* was swapped with the *asaC* ortholog from *A. sclerotiorum* (*asaC_AS*). Wild-type *A. flavu*s and the *A. flavus* CA14 control produce the same mix of ferriaspergillin (**3**) and its analogs (**Figures 3A, B**) on AFPA medium (see also **Figure S9, S10**). *A. sclerotiorum* produces ferrineoaspergillin (**4**, **Figure 3C** and **Figure S11**). The *A. flavus* strain swapped with the *asaC_AS* ortholog produces ferrineoaspergillin (**Figure 3D** and **Figure S12**) and a small shoulder peak with similar retention time to **3**. However, the identity of this shoulder peak could not be verified. These findings suggest that the two amino acid precursor residues forming the pyrazinone intermediate – and, subsequently, (neo)aspergillic acid and ferri(neo)aspergillin – are dependent only on AsaC.

### AsaC is solely responsible for the biosynthesis of aspergillic acid precursor pyrazinones

**Figure 4:**
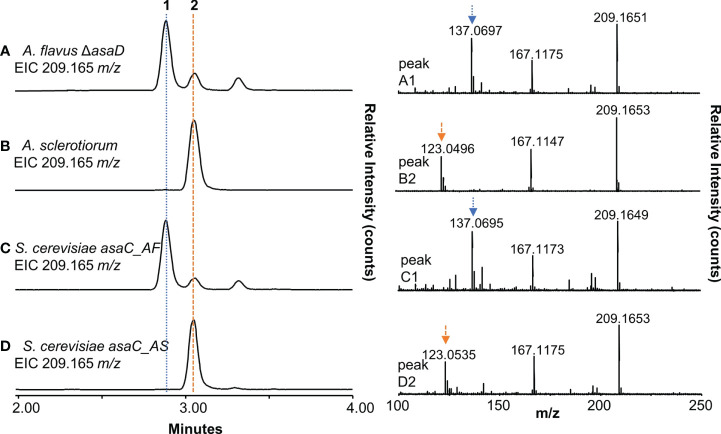
AsaC forms aspergillic acid precursor pyrazinones. **(A)**, left) Extracted ion chromatogram (EIC) of *A. flavus* Δ*asaD*. **(A)**, right) High energy mass spectra of peak 1 from *A. flavus* Δ*asaD* extract. **(B)**, left) EIC of *A. sclerotiorum* extract. **(B)**, right) High energy mass spectra of peak 2 from *A. sclerotiorum* extract. **(C)**, left) EIC of *S. cerevisiae* expressing *asaC* from *A. flavus* (*asaC_AF*) extract. **(C)**, right) High energy mass spectra of peak 1 from *S. cerevisiae asaC_AF* extract. **(D)**, left) EIC of *S. cerevisiae* expressing *asaC* from *A. sclerotiorum* (*asaC_AS*) extract. **(D)**, right) High energy mass spectra of peak 2 from *S. cerevisiae asaC_AS* extract. **1**: deoxyaspergillic acid; **2**: flavacol.

To further validate our observation that only the NRPS-like enzyme AsaC is necessary to selectively biosynthesize pyrazinones, we cloned *asaC_AF* and *asaC_AS* into yeast host strains and compared the yeast extracts to extracts of *A. flavus* Δ*asaD* and *A. sclerotiorum*. We previously reported that *A. flavus* Δ*asaD*, a knockout mutant of the P450 oxidase responsible for the conversion of deoxyaspergillic acid to aspergillic acid, produces an abundance of deoxyaspergillic acid (Lebar et al., 2018). Both deoxyaspergillic acid and flavacol share the molecular formula C_12_H_20_N_2_O and their [M+H]^+^ ion is 209.16484 *m/z*. The extracted ion chromatogram (EIC, *m/z* = 209.165) of *A. flavus* Δ*asaD* grown in yeast extract (YE) glycerol broth confirms that deoxyaspergillic acid (**1**) is present (**Figure 4A**, left and **Figure S13**), as is a small amount of flavacol (**2**, **Figure S14**). High energy MS data shows a distinct fragment peak at 137 m/z for deoxyaspergillic acid (**Figure 4A**, right). The extracted ion chromatogram (EIC, *m/z* = 209.165) of *A. sclerotiorum* grown in YE glycerol shows only one peak, flavacol (**2**), which has a distinct fragment at 123 *m/z* in its high energy MS spectrum (**Figure 4B** and **Figure S15**). The extract prepared from *S. cerevisiae* expressing *asaC* from *A. flavus* (*asaC_AF*) shows deoxyaspergillic acid (**1**) as the main product (**Figure 4C** and **Figure S16**), as well as a small amount of flavacol (**2**, **Figure S17**), while *S. cerevisiae* expressing *asaC* from *A. sclerotiorum* (*asaC_AS*) contains only flavacol (**2**, **Figure 4D** and **Figure S18**), mirroring their cognate fungal extracts. It has been previously shown that deoxyaspergillic acid is formed from one leucine and one isoleucine residue (MacDonald, 1961) and flavacol is formed from two leucine residues (Micetich and MacDonald, 1965). We herein show that AsaC selectively dictates which amino acids the pyrazinone products are derived from. AsaC_AF predominately selects isoleucine and leucine, while AsaC_AS selects two leucine residues. Previously reported NRPS-like enzymes that are comprised of one ATR module produce products comprised of two of the same amino acid (Forseth et al., 2013; Morgan et al., 2021), much like AsaC_AS. AsaC_AF is unique in that it makes a product with two distinct amino acids from one ATR module. It is also interesting to note that the pattern of relative amounts of deoxyaspergillic acid, flavacol, and a minor flavacol analog (RT = 3.32 min) in the *A. flavus* Δa*saD* strain and the yeast expressing *asaC_AF* are nearly identical (**Figure 4A, C**, see also **Figures S19, S20**). We can infer that this ratio is entirely controlled by the AsaC enzyme and is not dependent on any other genes in the *asa* cluster.

### The nonribosomal codes of AsaC in *Aspergillus* section *Flavi* and section *Circumdati* differ by only one residue

**Figure 5:**
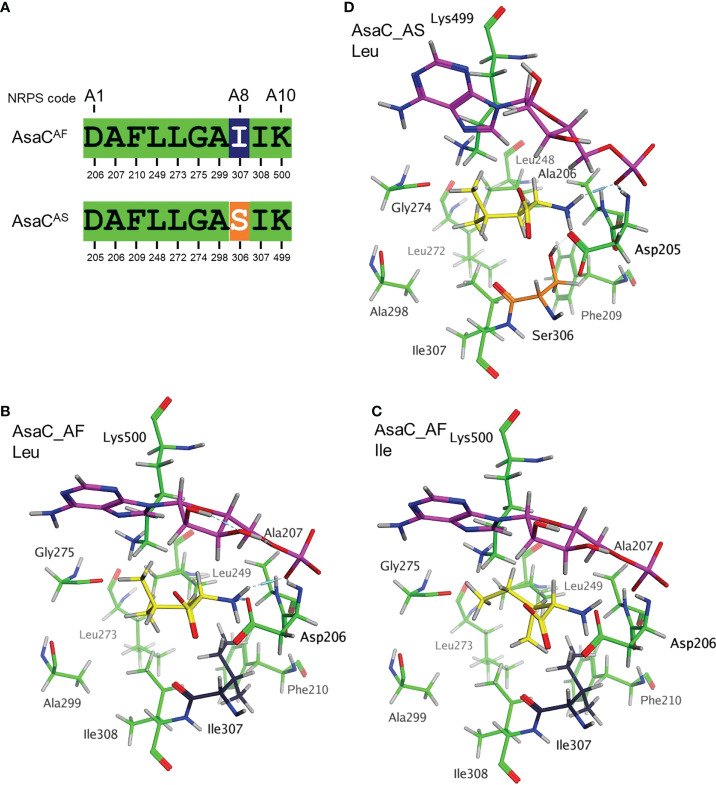
The nonribosomal codes of AsaC in *Aspergillus* section *Flavi* and section *Circumdati* differ by only one residue. **(A)** Alignment of the 10 key amino acid residues in the nonribosomal code using GrsA-PheA as a reference. AsaC_AF represents the code from *A. flavus* as well as AsaC from all *Aspergillus* section *Flavi* species sequenced to date (see **Figure 1**). AsaC_AS represents the code from *A. sclerotiorum* and AsaC from all *Aspergillus* section *Circumdati* species sequenced to date (see **Figure 1**). Stick rendering model of the 10 key amino acids in AsaC_AF **(B, C)** and AsaC_AS **(D)** enzymes using the PheA crystal structure (pdb 1AMU) as a template. Key amino acid colors are consistent with the alignment in panel **(A)**. Complexed amino acids leucine **(B, D)** or isoleucine **(C)** are colored yellow, and cAMP (in magenta) is orientated similarly near the top of each panel.

The adenylation (A) domains of NRPS enzymes selectively activate amino acids, which are then tethered to the thiolation (T) domain. The selectivity of the adenylation domain has been investigated extensively in bacteria and to a lesser extent, in fungi (Kudo et al., 2019). A seminal study reporting the crystal structure of the adenylation domain of the NRPS responsible for phenylalanine activation in gramicidin S biosynthesis (GrsA-PheA) identified ten key residues that are responsible for the selectivity (Conti et al., 1997). These ten residues became known as the “nonribosomal code” and are designated A1-A10. Alignment of the GrsA-PheA to the *Aspergillus* spp. AsaC protein sequences revealed that *Aspergillus* section *Flavi* and section *Circumdati* species share 9 of the 10 nonribosomal code residues (**Figure 5A**, **Figures S21, S22**). All section *Flavi* species contain isoleucine at A8, while all section *Circumdati* species contain serine at this position. To highlight the ten key amino acids in the A domains of AsaC_AF and AsaC_AS, model structures were prepared using the PheA (1AMU pdb) crystal as a template (Conti et al., 1997). The AsaC_AF and AsaC_AS models complexed with leucine or isoleucine were prepared by exchanging the substrate phenylalanine in the PheA crystal and preserving the orientation of the respective substrate amino acids. The models predict that both the highly conserved A1 aspartic acid and the A10 lysine residues lie proximal to the substrate Leu or Ile residues in AsaC_AF and AsaC_AS (**Figures 5B** **–D**). In each model, ionic interactions are predicted to form between the substrate leucine or isoleucine amino acid and the A1 aspartic acid consistent with interactions observed in the PheA (1AMU pdb) template. The highly conserved A10 lysine, along with glycine 275 (A6) and isoleucine 307 (A8) in AsaC_AF and glycine 274 (A6) and serine 306 (A8) in AsaC_AS are also predicted to interact with the substrate amino acids, but these interactions are predicted to be relatively much weaker in this set of models (**Figures 5B** **–D**).

## Discussion

**Figure 6:**
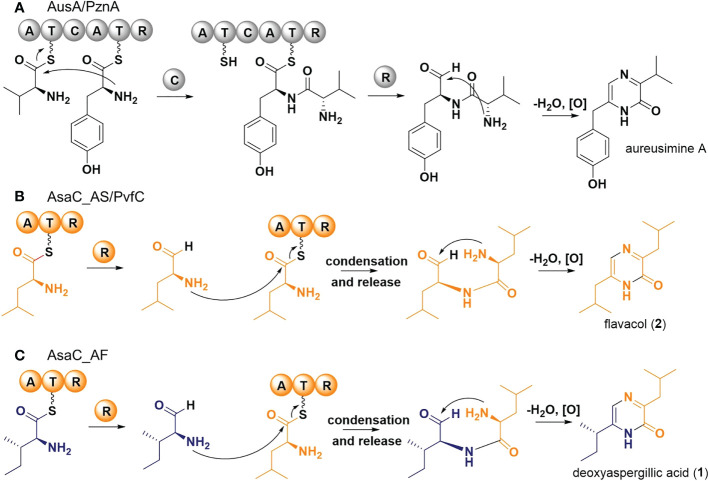
Proposed biosynthetic pathways of pyrazinone metabolites. **(A)** Schematic of the biosynthesis of aureusimine from valine and tyrosine by *Staphylococcus aureus* AusA. Adapted from (Wyatt et al., 2010; Zimmermann and Fischbach, 2010). **(B)** Schematic of the biosynthesis of flavacol (**2**) from two leucines by PvfC in *Pseudomonas fluorescens* and AsaC_AS in *Aspergillus sclerotiorum*. Adapted from (Morgan et al., 2021). **(C)** Schematic of the biosynthesis of deoxyaspergillic acid (**1**) from isoleucine and leucine by AsaC_AF in *A flavus*.

Typical NRPS enzymes are modular; each module (usually ATC) adds one distinct amino acid to the peptide chain (Schwarzer et al., 2003; Evans, 2016). Because pyrazinone metabolites arise from two amino acids, a dimodular NRPS would be predicted to form these products. Pyrazinone metabolites have been reported from bacteria, such as the aureusimines (=phevalin, tyrvalin) produced by *Staphylococcus aureus* (Wyatt et al., 2010; Zimmermann and Fischbach, 2010). AusA (=PzaA), a dimodular NRPS responsible for the biosynthesis of aureusimines, is comprised of the domains ATCATR. Each module (ATC and ATR) is responsible for activating a distinct amino acid, while the C forms a peptide bond between the two activated amino acids. The AusA reductase domain (R) liberates the dipeptide as an aldehyde and, after spontaneous cyclization and oxidation, the substituted pyrazinones are formed (**Figure 6A**) (Wilson et al., 2013).

Unexpectedly, and in contrast to the dimodular NRPS AusA (ATCATR) from *Staphylococcus*, the unimodular NRPS-like enzyme AsaC (ATR) forms pyrazinones in *Aspergillus* species. A unimodular ATR enzyme with similar activity from *Pseudomonas fluorescens* has been recently characterized. A detailed mechanistic investigation was conducted on PvfC by Li and coworkers (Morgan et al., 2021). PvfC is the core biosynthesis gene in the *Pseudomonas* virulence factor (*pvf*) biosynthetic gene cluster. PvfC was shown to make flavacol and a related substituted imidazole from two leucine residues. AsaC_AS likely functions similarly to the proposed mechanism for PvfC (**Figure 6B**): 1. A leucine residue is activated and tethered to the thiolation domain (T); 2. The reductase domain (R) liberates the residue as an aldehyde (NH_2_-Leu-CHO); 3. Another leucine residue is activated and tethered to T; 4. The amine from NH_2_-Leu-CHO formed in step 2 is condensed with the tethered leucine, liberating a dipeptide aldehyde (NH_2_-Leu-Leu-CHO); 5. Spontaneous cyclisation and oxidation then form flavacol (**2**). How the condensation in step 4 is accomplished without a canonical condensation domain requires further investigation.

The mechanism of AsaC_AF from *A. flavus* is more interesting since the pyrazinone product is formed from two different amino acids. We propose AsaC_AF first activates an isoleucine residue. After reductive release forming NH_2_-Ile-CHO, a leucine is then activated and tethered to T. In a similar mechanism to AsaC_AS, AsaC_AF likely facilitates condensation and release forming NH_2_-Leu-Ile-CHO, which affords deoxyaspergillic acid (**1**) after cyclisation and oxidation (**Figure 6C**). The initial activation step of AsaC_AF appears to be more promiscuous than the activation of leucine by AsaC_AS because we observed both deoxyaspergillic acid and a small amount of flavacol (from NH_2_-Leu-Ile-CHO and NH_2_-Leu-Leu-CHO, respectively) in AsaC_AF experiments, but only flavacol in the AsaC_AS experiments. Deoxyaspergillic acid is the predominant AsaC_AF product, however, indicating the enzyme is surprisingly selective as each of the two distinct amino acids must be selected in the appropriate order to afford the mixed disubstituted product. This pyrazinone product contains an alpha-isobutyl group and a *sec*-butyl adjacent to the amide NH. If the proposed mechanism is correct and the order of activations were switched, the positions of the butyl groups would also be switched. Further investigation is required to determine the mechanism by which AsaC_AF incorporates different amino acids at different steps of deoxyaspergillic acid biosynthesis with only one ATR module.

The modeling presented here provides a coarse prediction of the arrangement of the crucial ten amino acids in the Aspergillus AsaC_AF and AsaC_AS enzymes thought to be involved in substrate recognition based upon the adenylation domain in the PheA-GrsA template structure. In the PheA structure, the highly conserved A1 (Asp235) and A10 (Lys517) residues interact directly with the substrate phenylalanine (Conti et al., 1997). For example, the A1 (Asp235) residue forms electrostatic interactions with the amino group of the substrate phenylalanine while the A10 (Lys517) residue interacts with substrate phenylalanine carboxylic acid. This is not necessarily the case in the *Aspergillus* AsaC models. In both Aspergillus AsaC enzymes the A1 (Asp205 or Asp206) residue is oriented so that it would be predicted to interact favorably with the amino group of the substrate amino acid. In contrast, the A10 (Lys499 or Lys500) is positioned in manner so that it generates electrostatic interactions with Gly274, but the carboxylic acid of the substrate amino acid is rotated away from the A10 lysine (**Figure 5**). As discussed above, the AsaC_AF and AsaC_AS enzymes have different substrate amino acid preferences. The A8 residues, Ile307 and Ser306, that differ between the two *Aspergillus* enzymes may play a role in substrate recognition (leucine or isoleucine) and therefore which pyrazinone products are produced. Ile307 from AsaC_AF and the Ser306 from AsaC_AS are positioned just below the respective A1 residues (Asp205 or Asp206) in the substrate pocket. These A8 residues are predicted to interact with the A1 residue(s) and this may allow them some influence on substrate specificity. Site-directed mutagenesis combined with further modeling of the *Aspergillus* enzymes will help to elucidate the structure-function relationship of specific amino acids for these ATR enzymes.

While the exact function of the pyrazinone natural products produced by *Aspergillus* species with respect to virulence is unknown, Dolezal et al. (2013) reported that *asaC* was upregulated during *A. flavus* infection of corn. We were later able to show that an *A. flavus* mutant in which *asaC* was knocked out was less virulent in a corn kernel infection model when compared to the control strain that harbored an intact *asaC* gene. Little is currently known about the function of these metabolites in *A. sclerotiorum* and other ochratoxin A-producing aspergilli. Because the downstream metabolites in the *asa* biosynthesis pathways are hydroxamic acid-containing pyrazinones that bind iron (e.g. aspergillic acid and neoaspergillic acid), it is quite possible that these molecules are acting as siderophores, thus contributing to *Aspergillus* virulence (Haas, 2014). Flavacol can be isolated from both *A. flavus* and *A. sclerotiorum* and could be acting as a signaling molecule for virulence, which has been reported in some bacteria. The *Pseudomonas* virulence factor (*pvf*) gene cluster, which includes the flavacol-producing ATR enzyme PvfC, is responsible for the production of unidentified autoinducers with many functions (Kretsch et al., 2021). PVF autoinducers were found to regulate a variety of secreted molecules, including toxins and proteins, that play a key role in bacterial virulence. Many pyrazinone-producing NRPSs have also been found in gut bacteria and appear to be involved in host colonization. NRPS-like enzymes have been engineered to make complex and diverse small molecules that resemble natural products (van Dijk et al., 2016; van Dijk and Wang, 2018).

## Conclusion

We have shown that AsaC NRPS-like enzymes with only an ATR module selectively produce disubstituted pyrazinones. Both AsaC homologs from *A. flavus* and *A. sclerotiorum* lack a C domain but are able to condense two amino acids to produce substituted pyrazinones. The selectivity of AsaC_AF from *A. flavus* is especially interesting in that it selectively forms the dipeptide product from two different amino acids while harboring only one A domain. Because these small genes produce functional enzymes when cloned into yeast, their selectivity can be further probed and, ideally, modified to produce a wide variety of substituted pyrazinone products. Further harnessing the biosynthetic machinery that produces these simple yet abundant and important pyrazinone natural products could lead to treatment strategies that have the potential to encourage beneficial microbes or inhibit pathogenic microbes in a variety of hosts.

## Data availability statement

The original contributions presented in the study are included in the article/**Supplementary Material**. Further inquiries can be directed to the corresponding author.

## Author contributions

ML and JC designed the research. ML, JC, BM, CC-W, QW, and CM performed experiments and analysed data. All authors wrote the manuscript and approved the submitted version.

## Conflict of interest

The authors declare that the research was conducted in the absence of any commercial or financial relationships that could be construed as a potential conflict of interest.

## Publisher’s note

All claims expressed in this article are solely those of the authors and do not necessarily represent those of their affiliated organizations, or those of the publisher, the editors and the reviewers. Any product that may be evaluated in this article, or claim that may be made by its manufacturer, is not guaranteed or endorsed by the publisher.
