# Supplementary material for: Small NRPS-like enzymes in Aspergillus sections Flavi and Circumdati selectively form substituted pyrazinone metabolites
Source: Front Fungal Biol. 2022 Oct 26;3:1029195. doi: 10.3389/ffunb.2022.1029195 (PMC10512218; doi:10.3389/ffunb.2022.1029195)
Supplement: Supplementary file 1 [file DataSheet_1.pdf]

Supplementary Material

Table S1. Genomes used for AsaC tree (see spreadsheet Table_S1.xlsx)
Figure S1. Plasmid used to generate swap mutant
Figure S2. Recombinant plasmid for swap (NRPS-3)
Figure S3. *Bam*HI restriction enzyme digestion of recombinant plasmid (NRPS-3)
Figure S4. Swap mutant transformants confirmed by PCR
Figure S5. Swap mutant transformants confirmed by PCR (continued)
Figure S6. The *asaC_AS* gene from *A. sclerotiorum*
Figure S7. Map of plasmid XW55-asaC_AS
Figure S8. Restriction enzyme digestion of the plasmid expressing *asaC_AS* in yeast
Figure S9. Supplemental data for ferriaspergillin (**3**) from *A. flavus* AF70 WT
Figure S10. Supplemental data for ferriaspergillin (**3**) from *A. flavus* CA14 *pyrG*+ control
Figure S11. Supplemental data for ferrineoaspergillin (**4**) from *A. sclerotiorum*
Figure S12. Supplemental data for ferrineoaspergillin (**4**) from *A. flavus* CA14 *swapped with asaC_AS*
Figure S13. Supplemental data for deoxyaspergillic acid (**1**) from *A. flavus* Δ*asaD* (peak A1)
Figure S14. Supplemental data for flavacol (**2**) from *A. flavus* Δ*asaD* (peak A2)
Figure S15. Supplemental data for flavacol (**2**) from *A. sclerotiorum* (peak B2)
Figure S16. Supplemental data for deoxyaspergillic acid (**1**) from *S. cerevisiae asaC_AF* (peak C1)
Figure S17. Supplemental data for flavacol (**2**) from *S. cerevisiae asaC_AF* (peak C2)
Figure S18. Supplemental data for flavacol (**2**) from *S. cerevisiae asaC_AS* (peak D2)
Figure S19. Supplemental data for flavacol analog from *S. cerevisiae asaC_AF* (peak A3)
Figure S20. Supplemental data for flavacol analog from *S. cerevisiae asaC_AS* (peak C3)
Figure S21. Nonribosomal code (A2-A9) of AsaC adenylation domain for all species in Figure 2
Figure S22. Alignment of AsaC to GrsA-PheA for all species in Figure 2

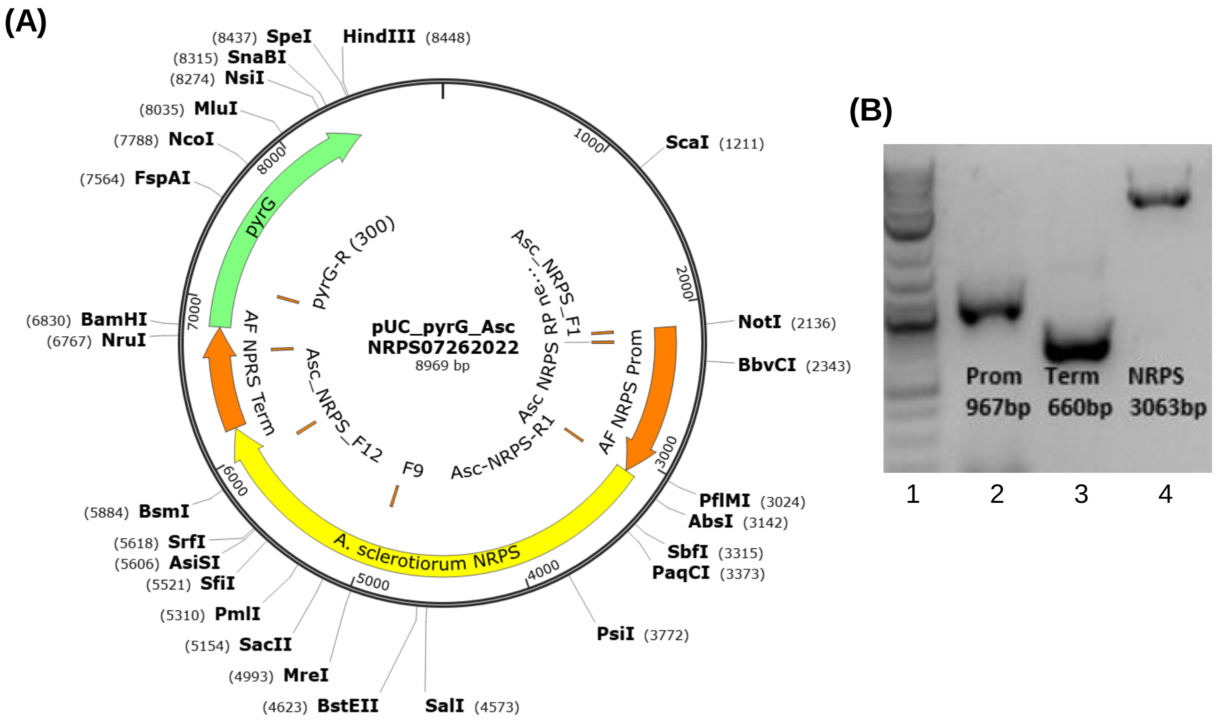

Figure S1. Plasmid used to generate swap mutant. (A) SeqBuilder map of plasmid. (B) PCR of native promoter and terminator for *asaC* DNA size markers (lane 1) and three amplified PCR fragments (lanes 2, 3, 4).

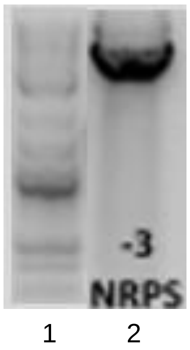

Figure S2. Recombinant plasmid (NRPS-3) for swap. Confirmed by PCR with primer pair Asc_NRPS_RP_nest_F and Asc_NRPS_RP_nest_R (see **Table 2**).

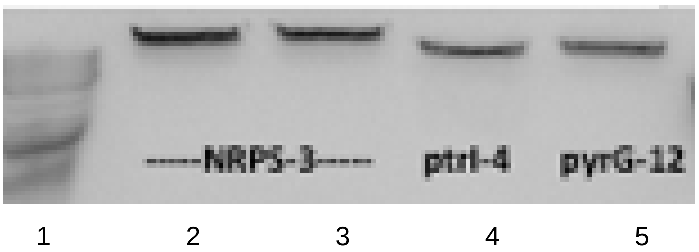

Figure S3. *Bam*HI restriction enzyme digestion of recombinat plasmid (NRPS-3). DNA size markers (lane 1) and BamHI-digested recombinant plasmid (NRPS-3) (about 9 kb) (lanes 2, 3) and two plasmid vectors (pUC19_ptrI-4 and pUC19_pyrG) (lanes 4, 5).

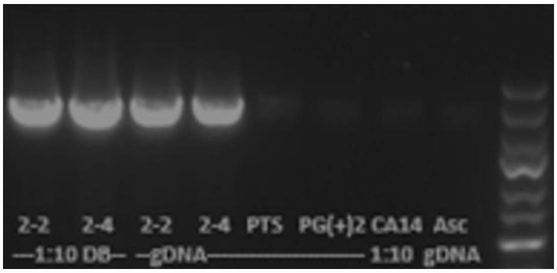

1 2 3 4 5 6 7 8

Figure S4. Swap mutant transformants confirmed by PCR. The 981 bp fragment from two transformants 2-2 and 2-4: direct PCR from mycelia (lanes 1, 2) or PCR using extracted gDNA (lanes 3, 4). Lanes 5, 6, 7, 8 are from PTS (parental strain), PG(+)2, CA14 (*A. flavus*), and Asc (*A. sclerotiorum*). The PCR primer pair used is a Asc_NRPS_F1 and Asc_NRPS_R1 (see **Table 2**).

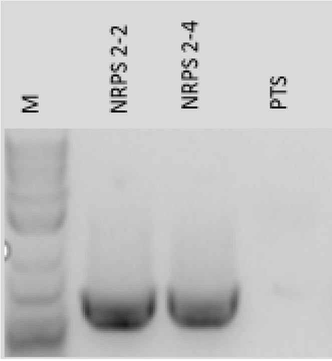

Figure S5. Swap mutant transformants confirmed by PCR (continued). The 1,206 bp fragment from two transformants 2-2 and 2-4 using PCR primers Asc_NRPS_F12 and pyrG-R (300) (see **Table 2**).

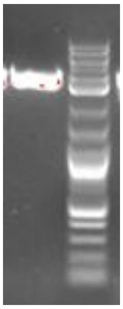

1 2

Figure S6. The *asaC_AS* gene from *A. sclerotiorum.* 3063 bp *asaC_AS* product was PCR amplified from *A. sclerotiorum* gDNA with primer pair Asc_023020_F and Asc_023020_R (lane 1). See **Table 2** for primers.

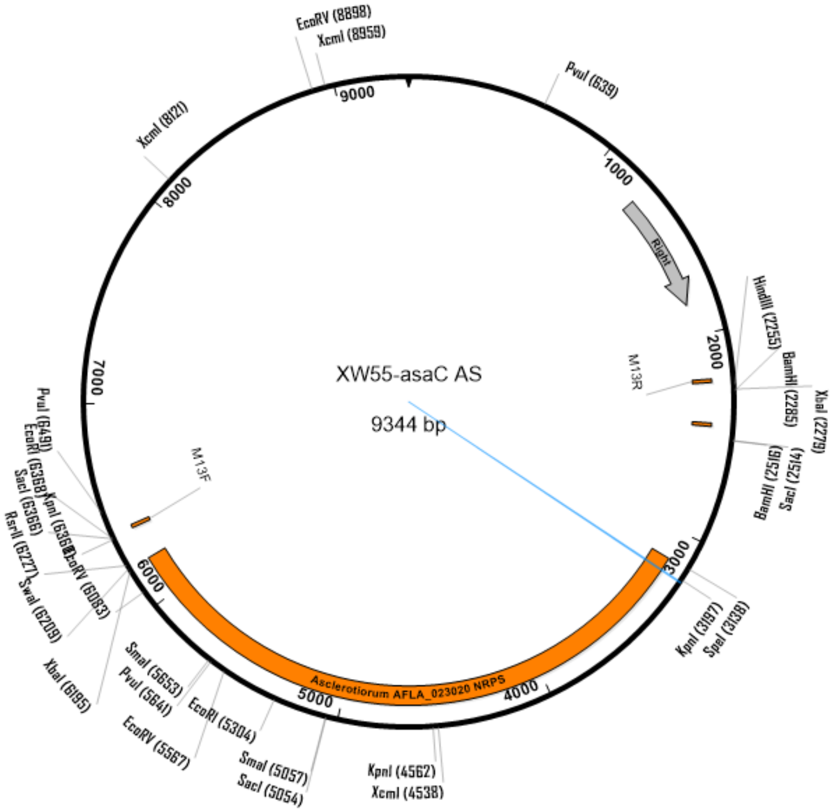

Figure S7. Map of plasmid XW55-asaC_AS

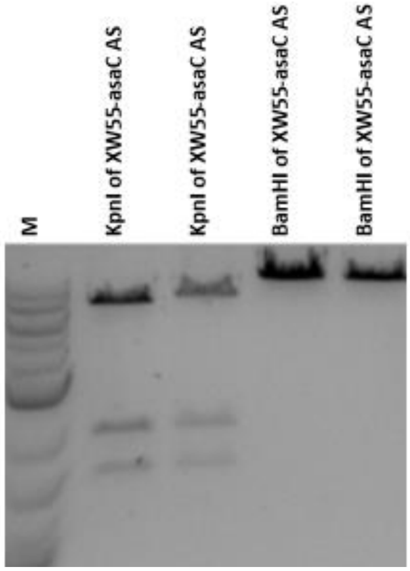

Figure S8. Restriction enzyme digestion of the plasmid expressing *asaC_AS* in yeast. Two recombinant plasmids confirmed by restriction digestion of KpnI (1.4 kb/1.8 kb/6.2 kb) and BamHI (9.1 kb).

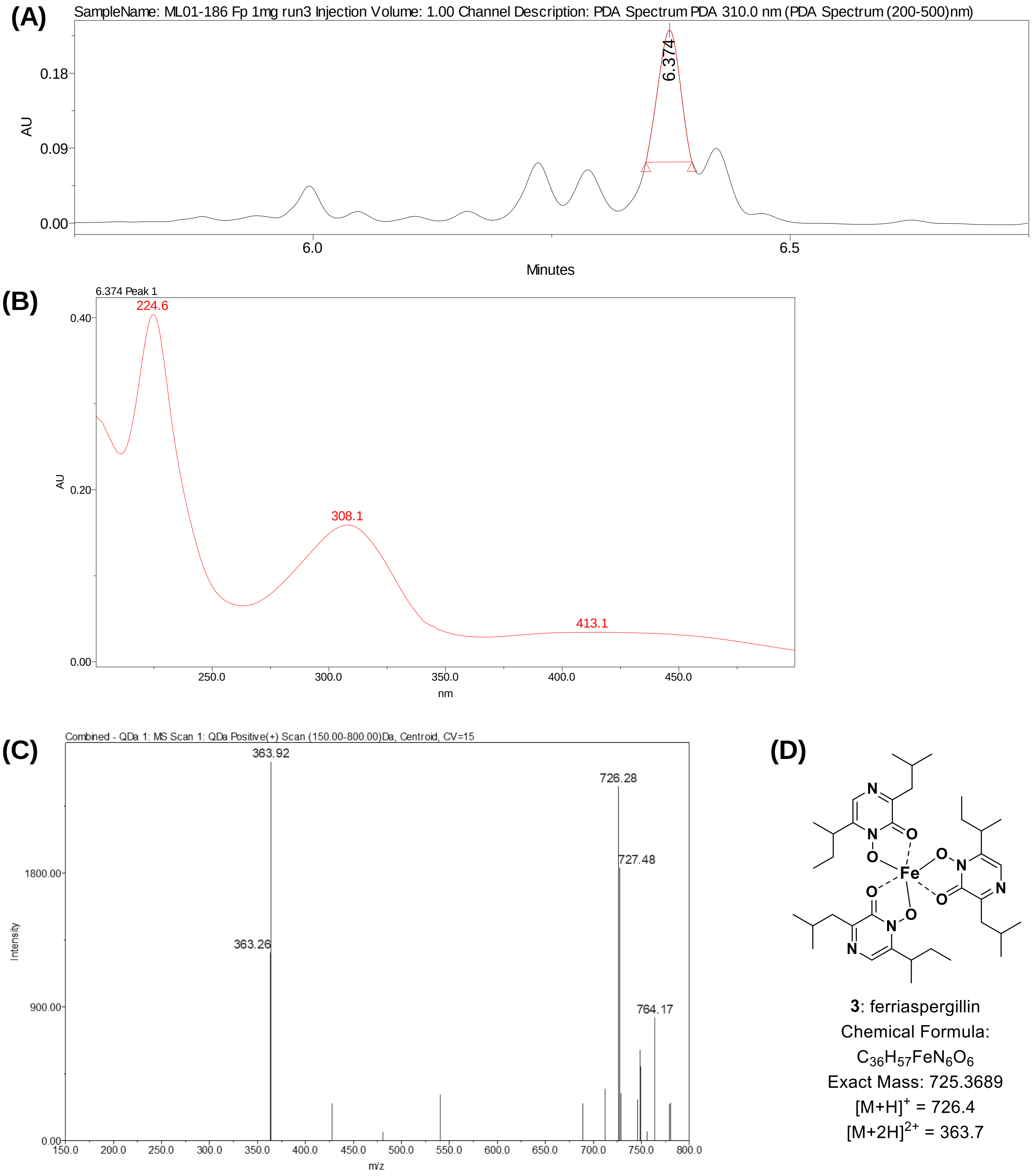

Figure S9. Supplemental data for ferriaspergillin (3) from *A. flavus* AF70 WT. (A) Chromatogram at λ = 310 nm, (B) UV spectrum, (C) mass spectrum, and (D) structure of ferriaspergillin (**3**) peak in Figure 3B.

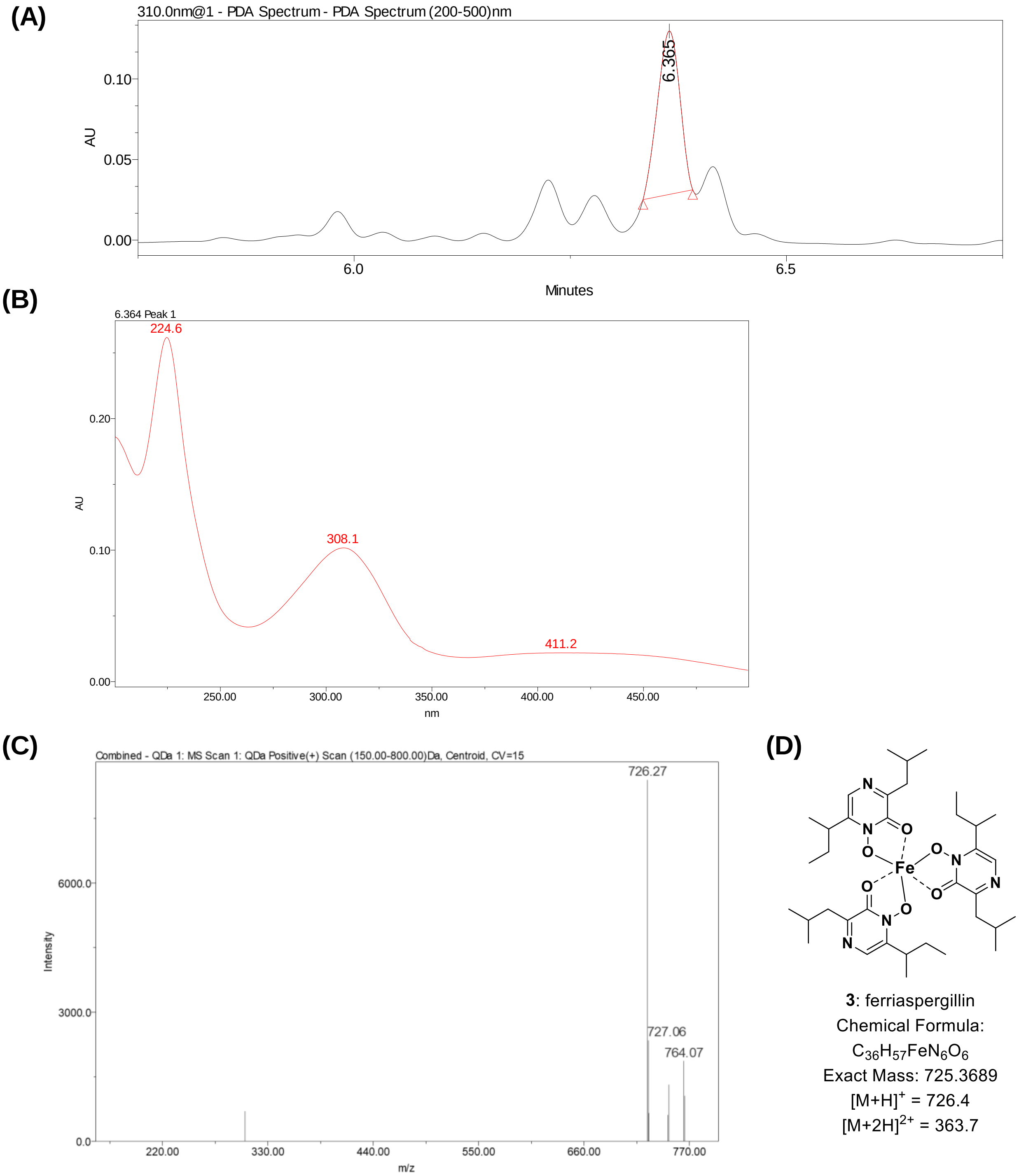

Figure S10. Supplemental data for ferriaspergillin (3) from *A. flavus* CA14 *pyrG+* control. (A) Chromatogram at λ = 310 nm, (B) UV spectrum, (C) mass spectrum, and (D) structure of ferriaspergillin (**3**) peak in Figure 3C.

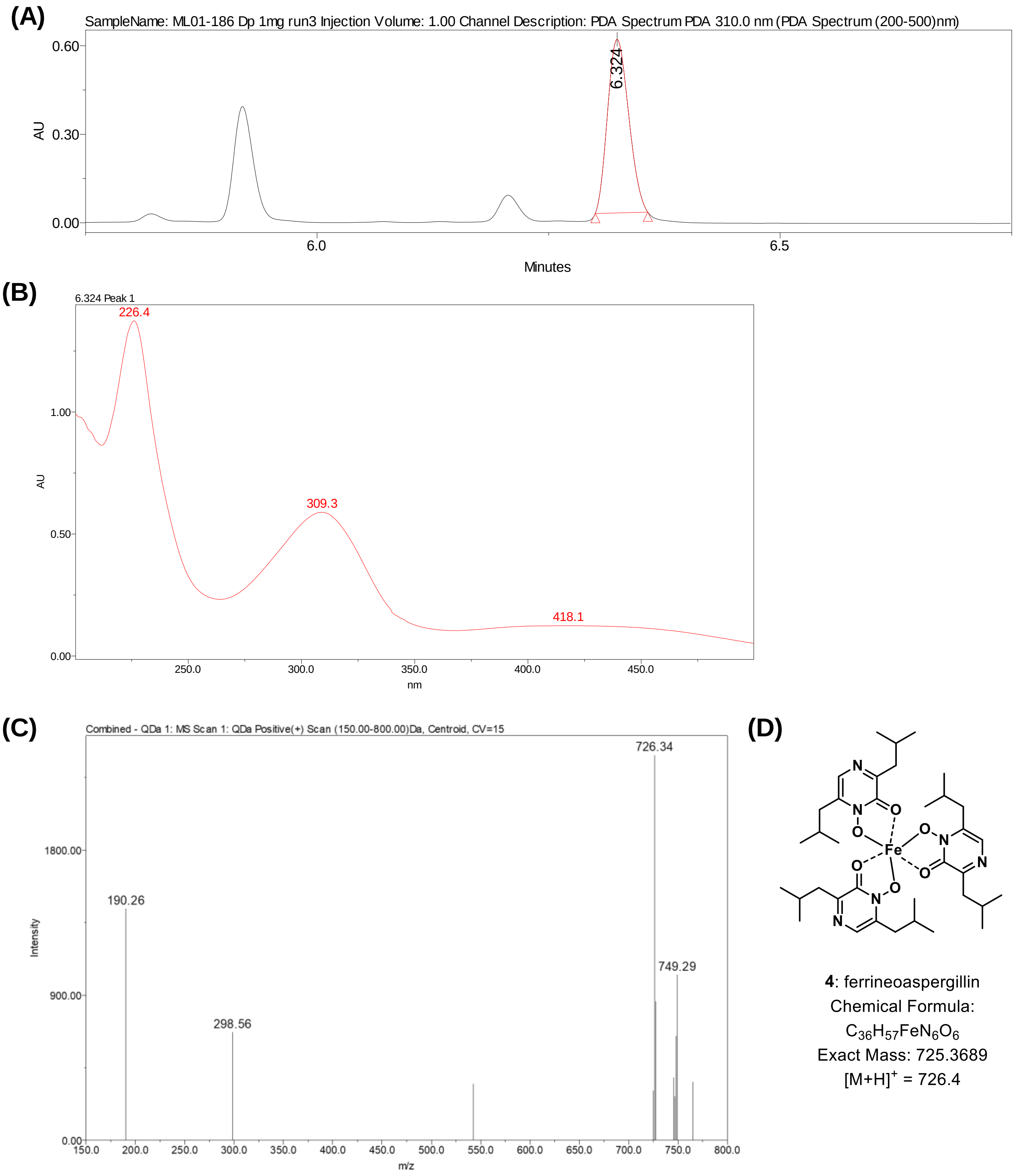

Figure S11. Supplemental data for ferrineoaspergillin (4) from *A. sclerotiorum*. (A) Chromatogram at λ = 310 nm, (B) UV spectrum, (C) mass spectrum, and (D) structure of ferrineoaspergillin (**4**) peak in Figure 3D.

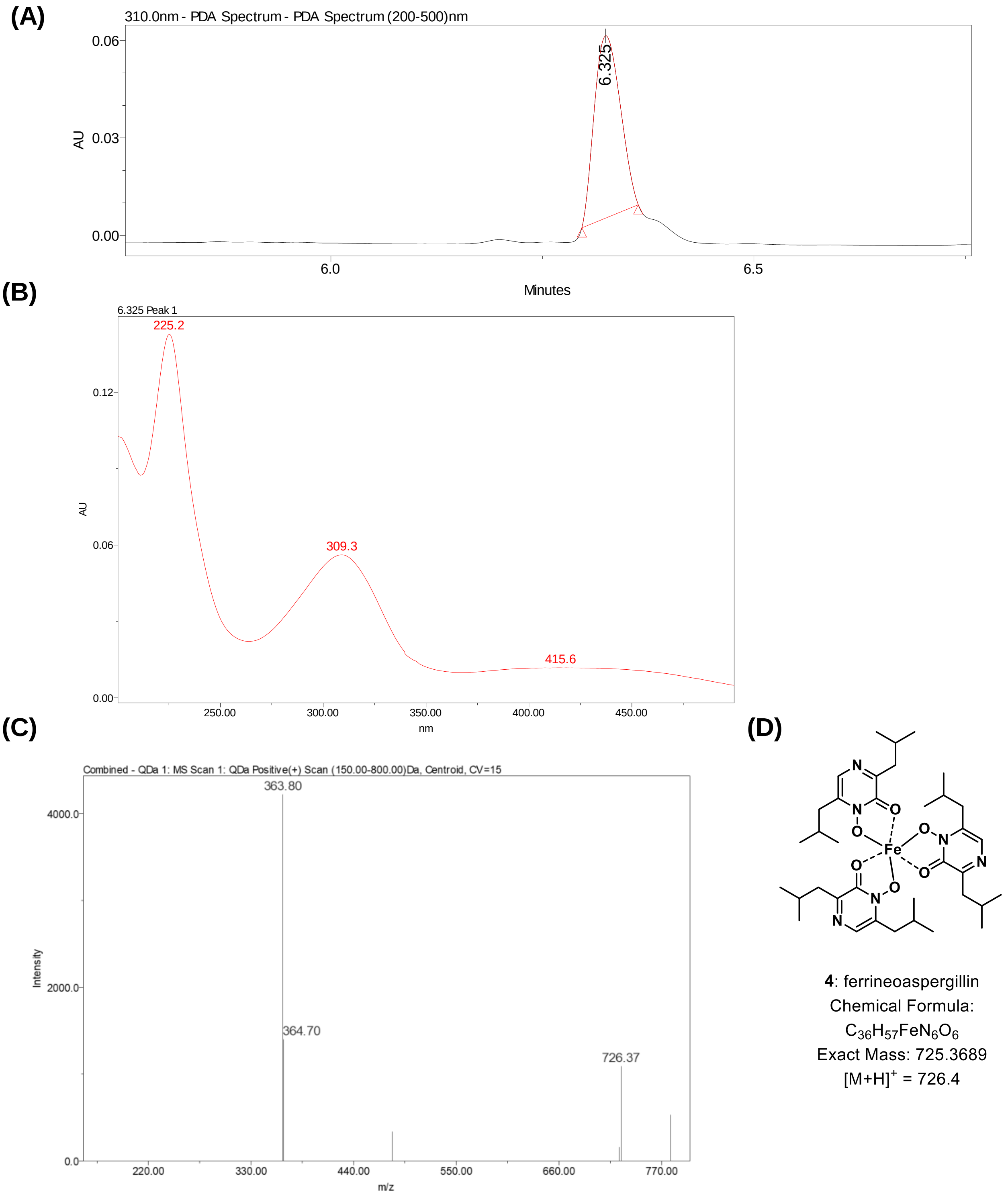

Figure S12. Supplemental data for ferrineoaspergillin (4) from *A. flavus* CA14 *swapped with asaC_AS*. (A) Chromatogram at λ = 310 nm, (B) UV spectrum, (C) mass spectrum, and (D) structure of ferrineoaspergillin (**4**) peak in Figure 3E.

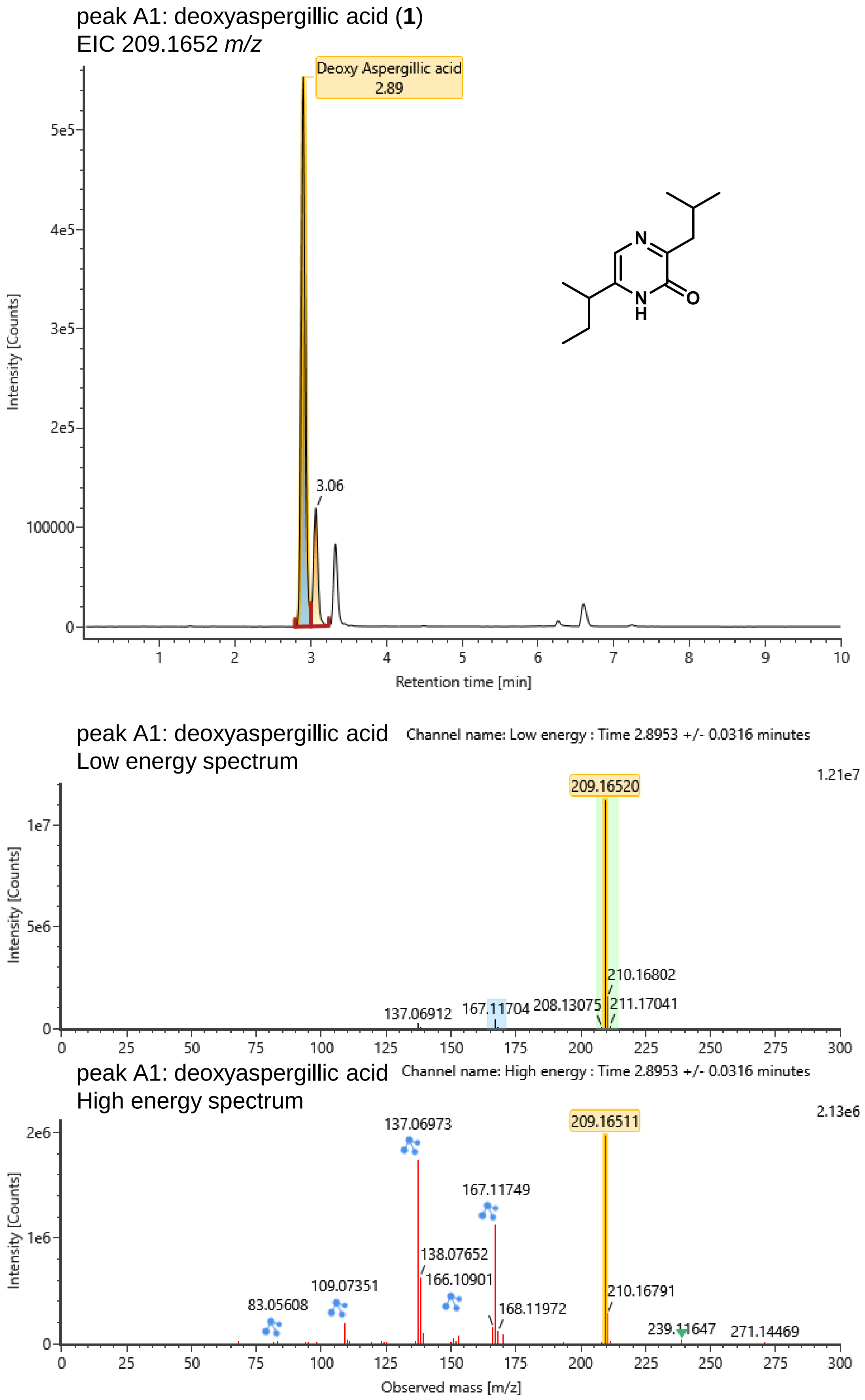

Figure S13. Supplemental data for deoxyaspergillic acid (**1**) from *A. flavus* Δ*asaD* (peak A1).

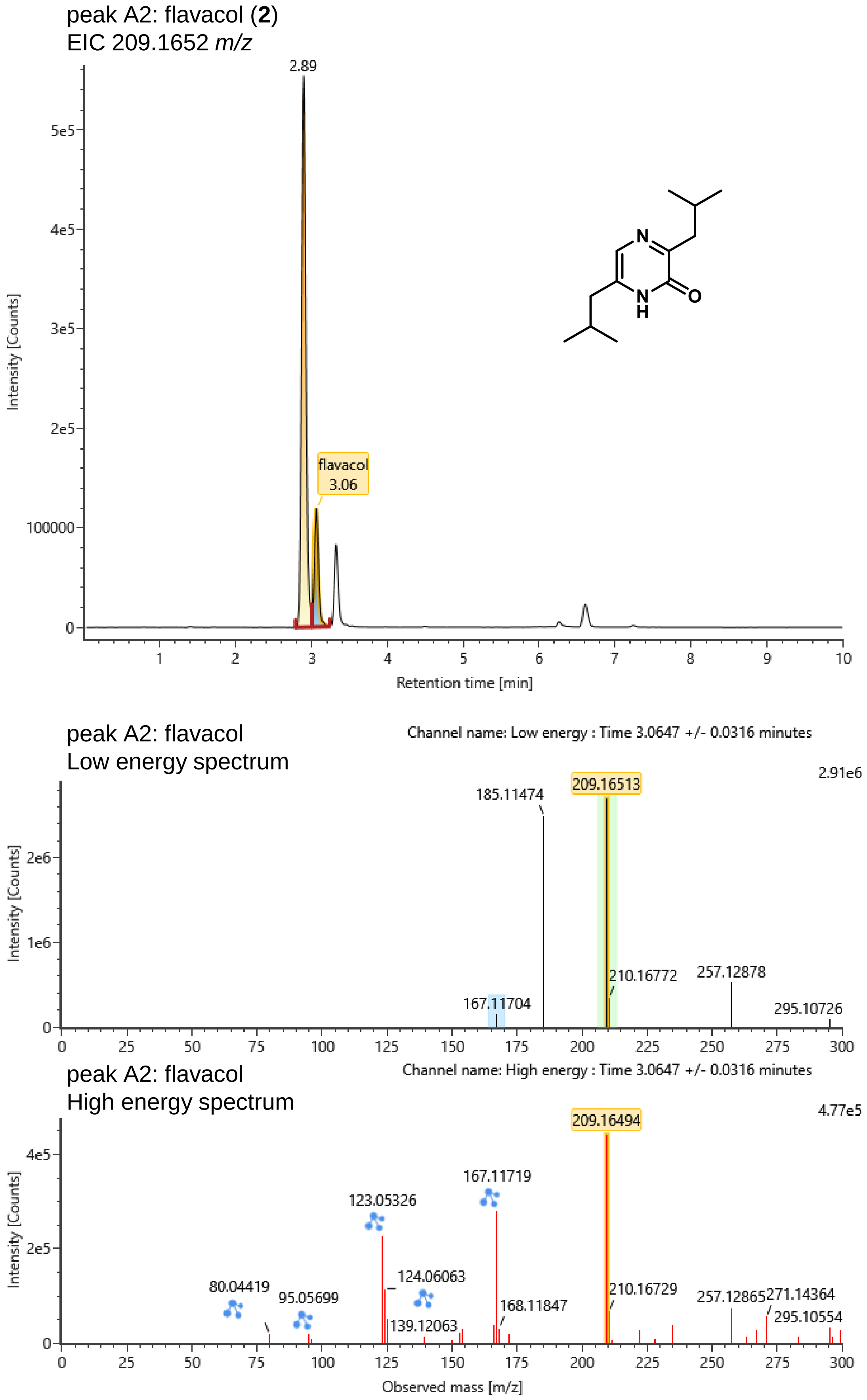

Figure S14. Supplemental data for flavacol (**2**) from *A. flavus* Δ*asaD* (peak A2).

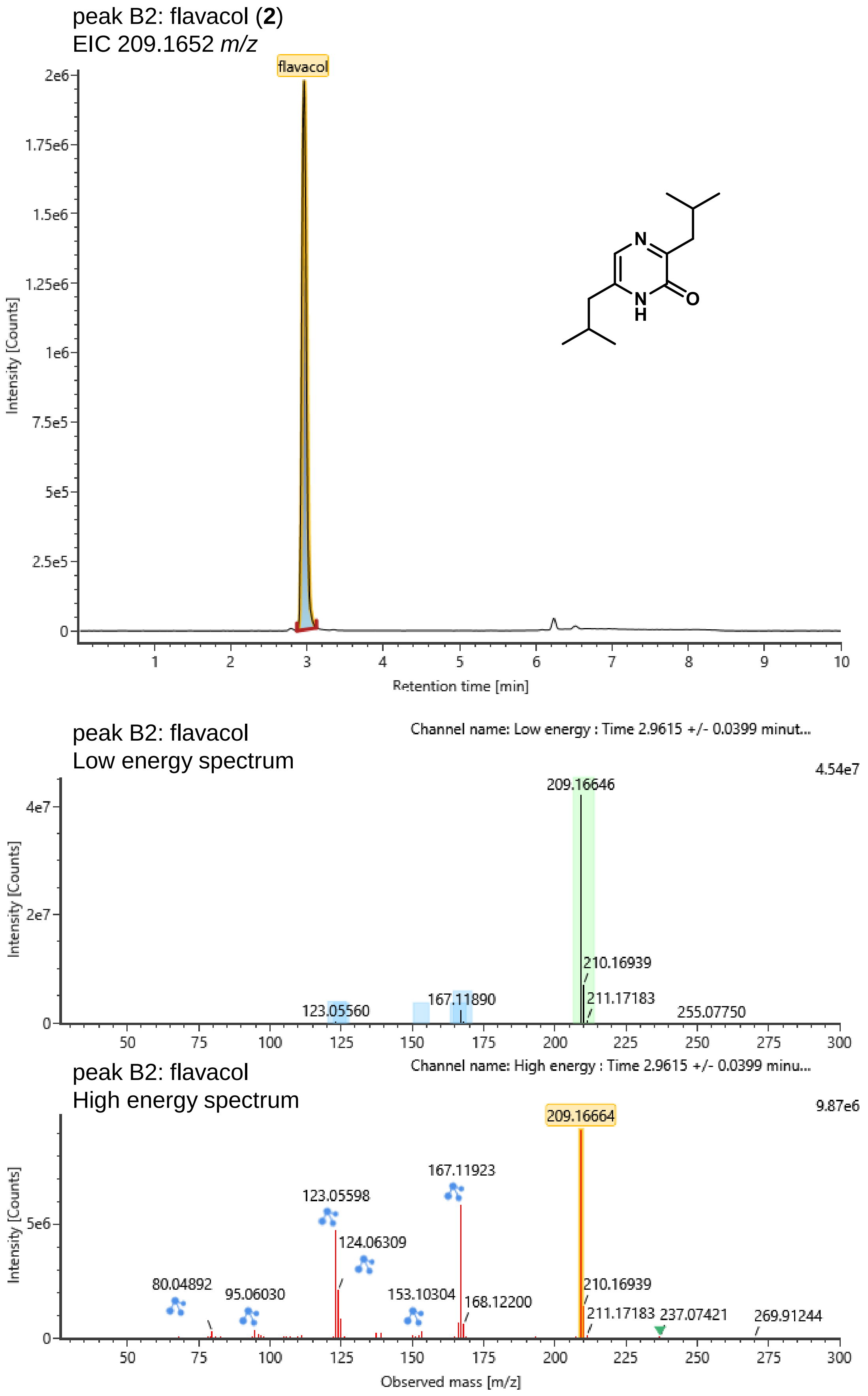

Figure S15. Supplemental data for flavacol (**2**) from *A. sclerotiorum* (peak B2).

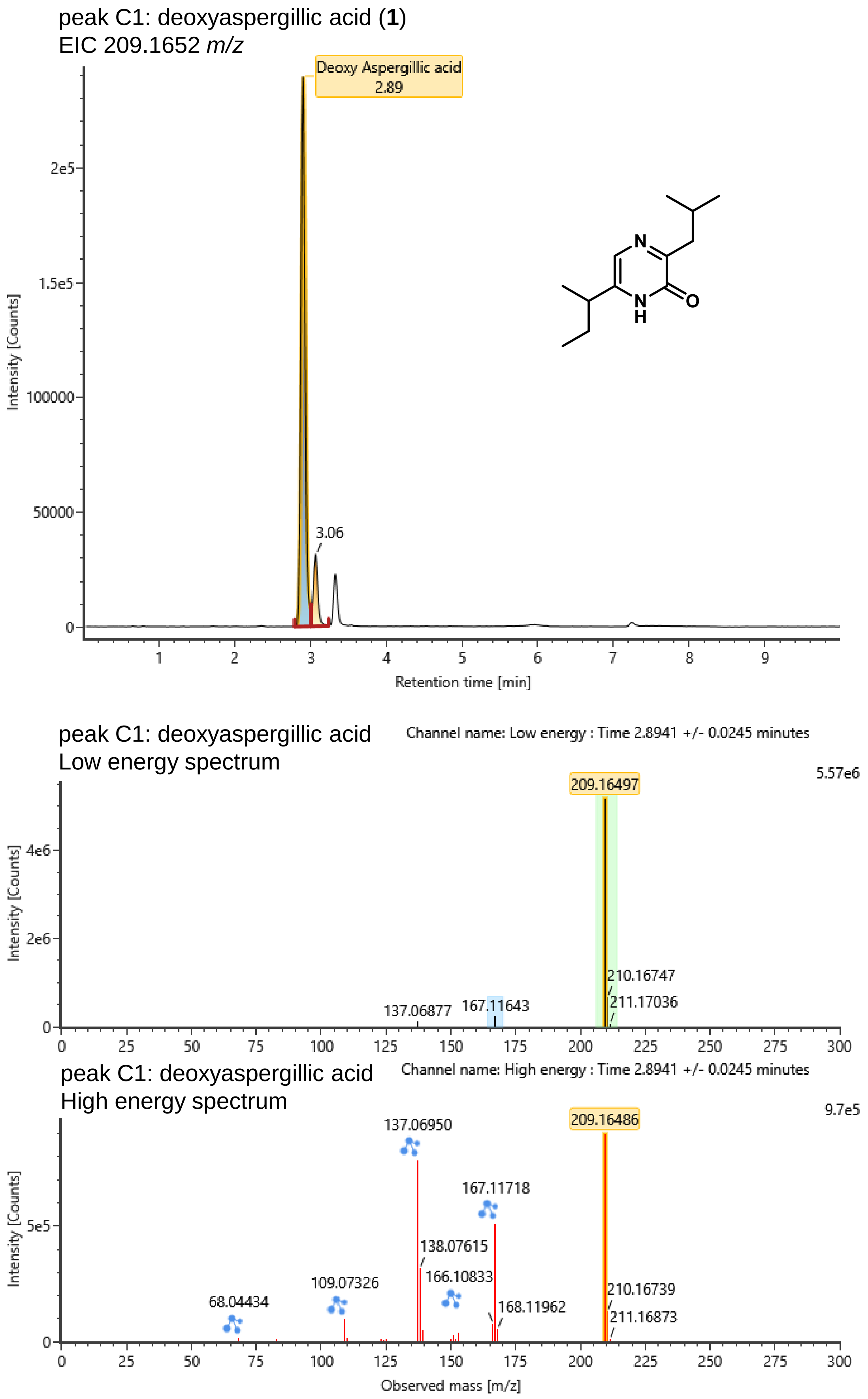

Figure S16. Supplemental data for deoxyaspergillic acid (**1**) from *S. cerevisiae asaC_AF* (peak C1).

peak C2: flavacol (**2**)
EIC 209.1652 *m/z*

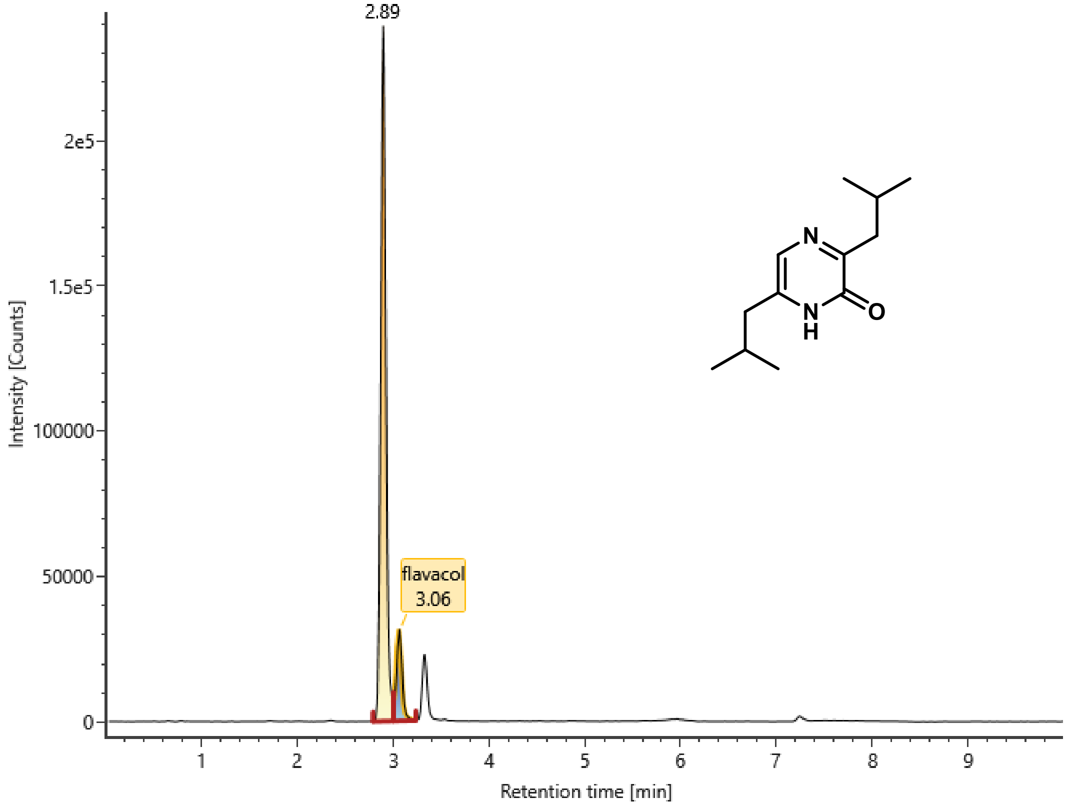

peak C2: flavacol (**2**)
Low energy spectrum

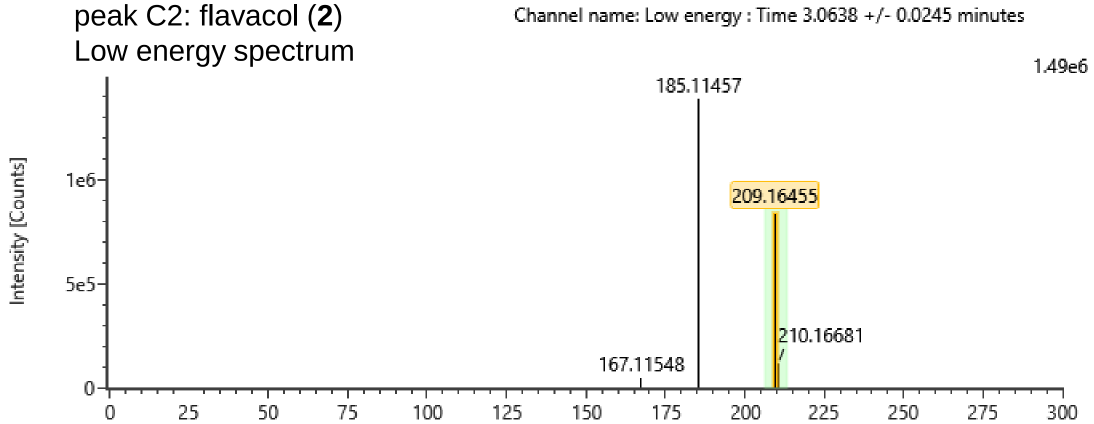

peak C2: flavacol (**2**)
High energy spectrum

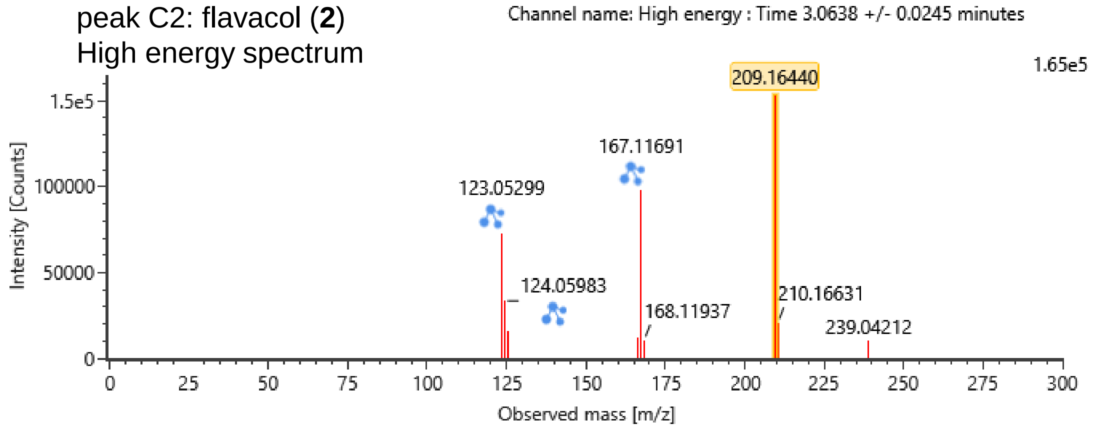

Figure S17. Supplemental data for flavacol (**2**) from *S. cerevisiae asaC_AF* (peak C2).

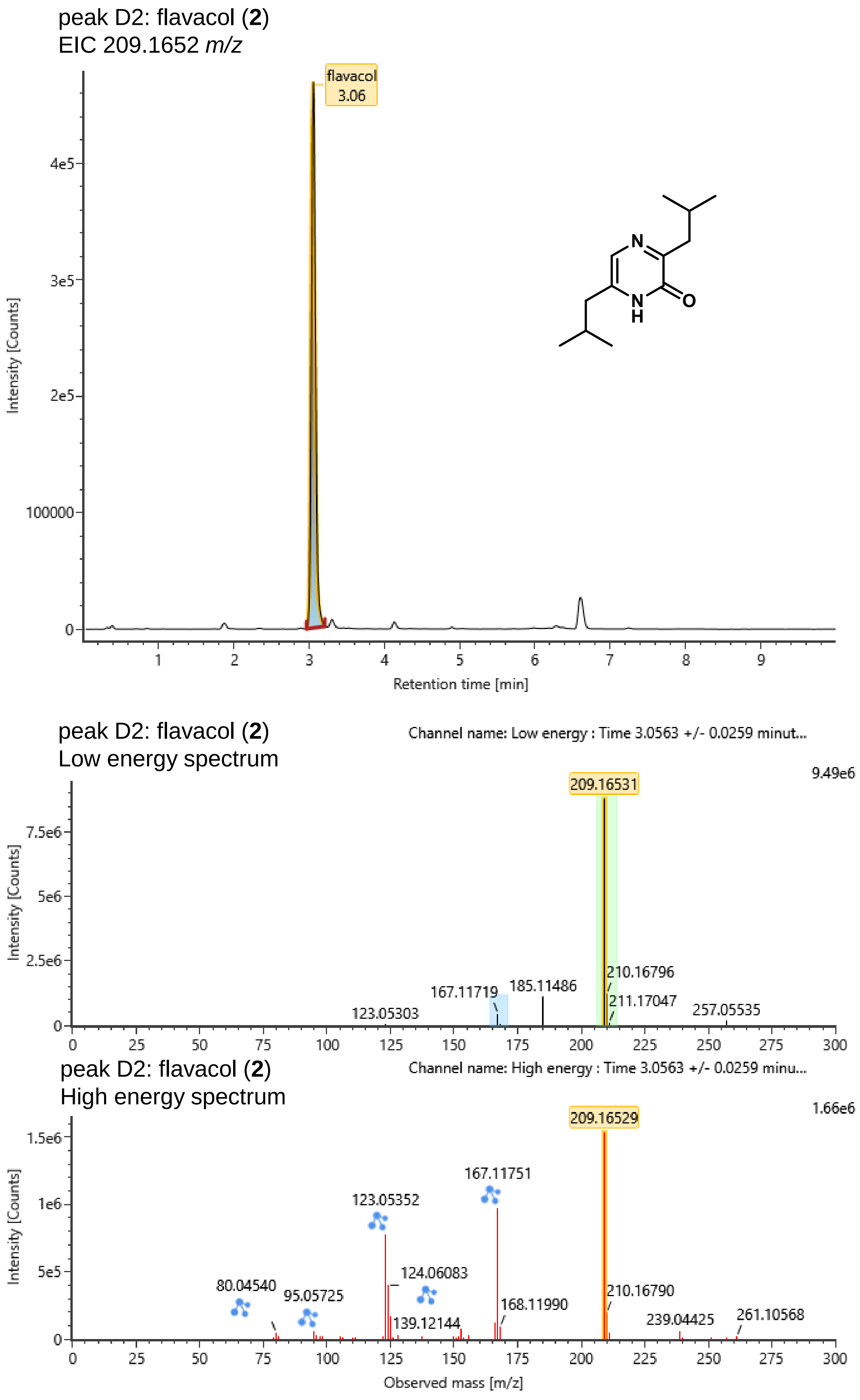

Figure S18. Supplemental data for flavacol (**2**) from *S. cerevisiae asaC_AS* (peak D2).

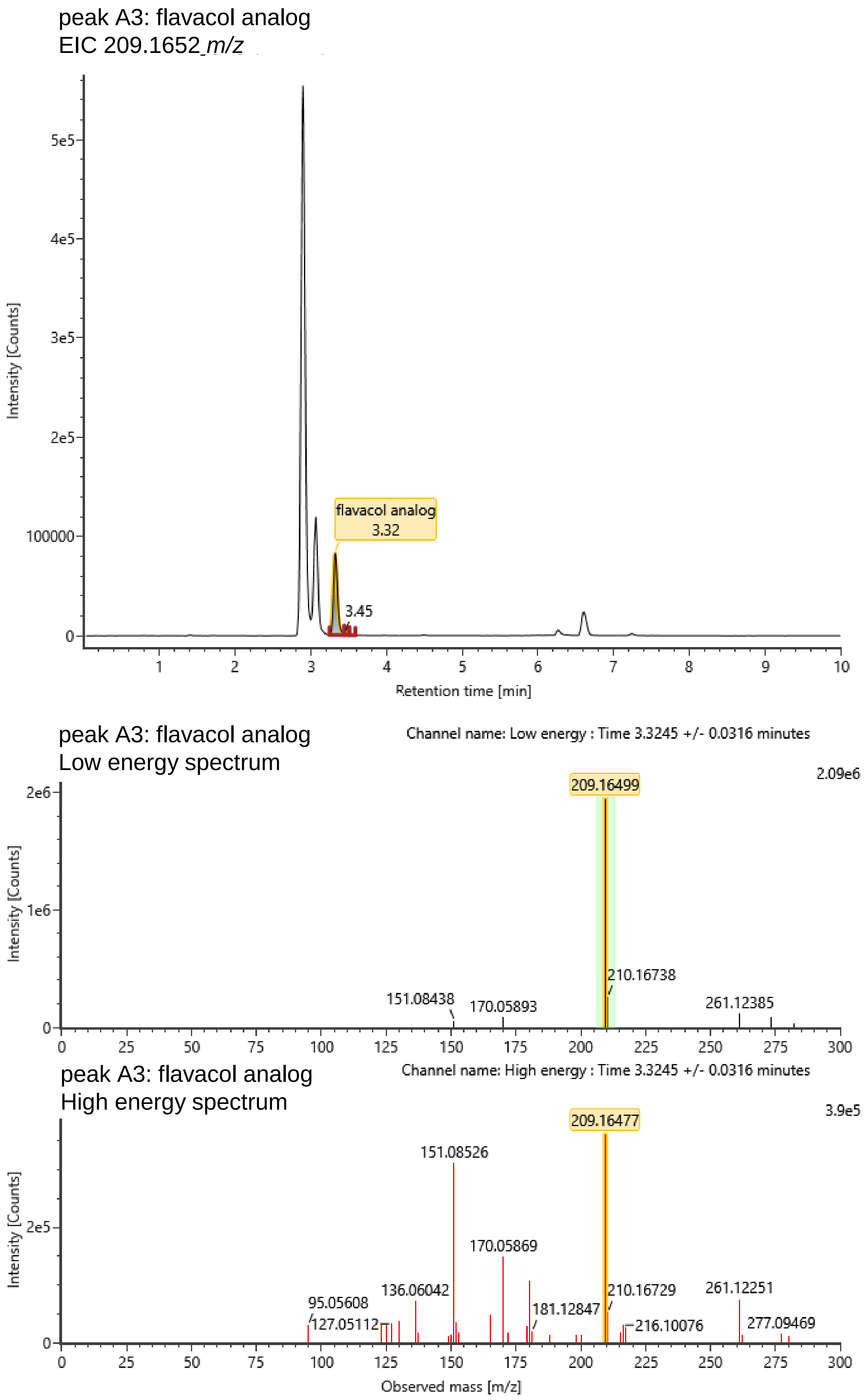

Figure S19. Supplemental data for flavacol analog from *S. cerevisiae asaC_AF* (peak A3).

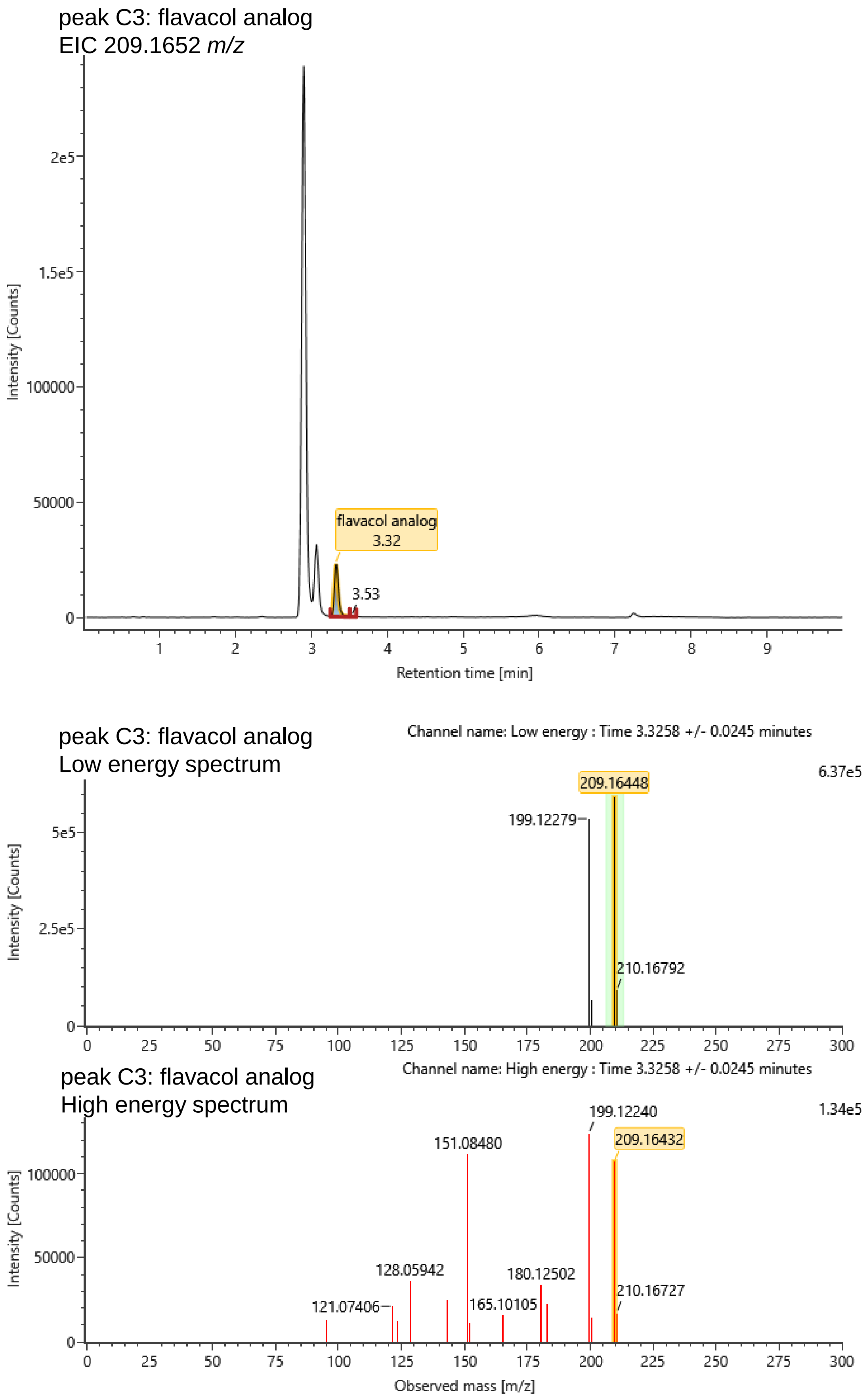

Figure S20. Supplemental data for flavacol analog from *S. cerevisiae asaC_AS* (peak C3).

Figure S21. Nonribosomal code (A2-A9) of AsaC adenylation domain for all species in Figure 2. All *Aspergillus* species were aligned to GrsA-PheA (see Data Sheet 2.pdf).

Figure S22. Alignment of AsaC to GrsA-PheA for all species in Figure 2. Nonribosomal code residues are indicated with brown squares. AsaC alignments contain all domains (ATR). The GrsA-PheA sequence shows only the A domain of GrsA (see Data Sheet 3.pdf).
