# Supplementary figures and images for: Small NRPS-like enzymes in Aspergillus sections Flavi and Circumdati selectively form substituted pyrazinone metabolites
Source: Front Fungal Biol. 2022 Oct 26;3:1029195. doi: 10.3389/ffunb.2022.1029195 (PMC10512218; doi:10.3389/ffunb.2022.1029195)

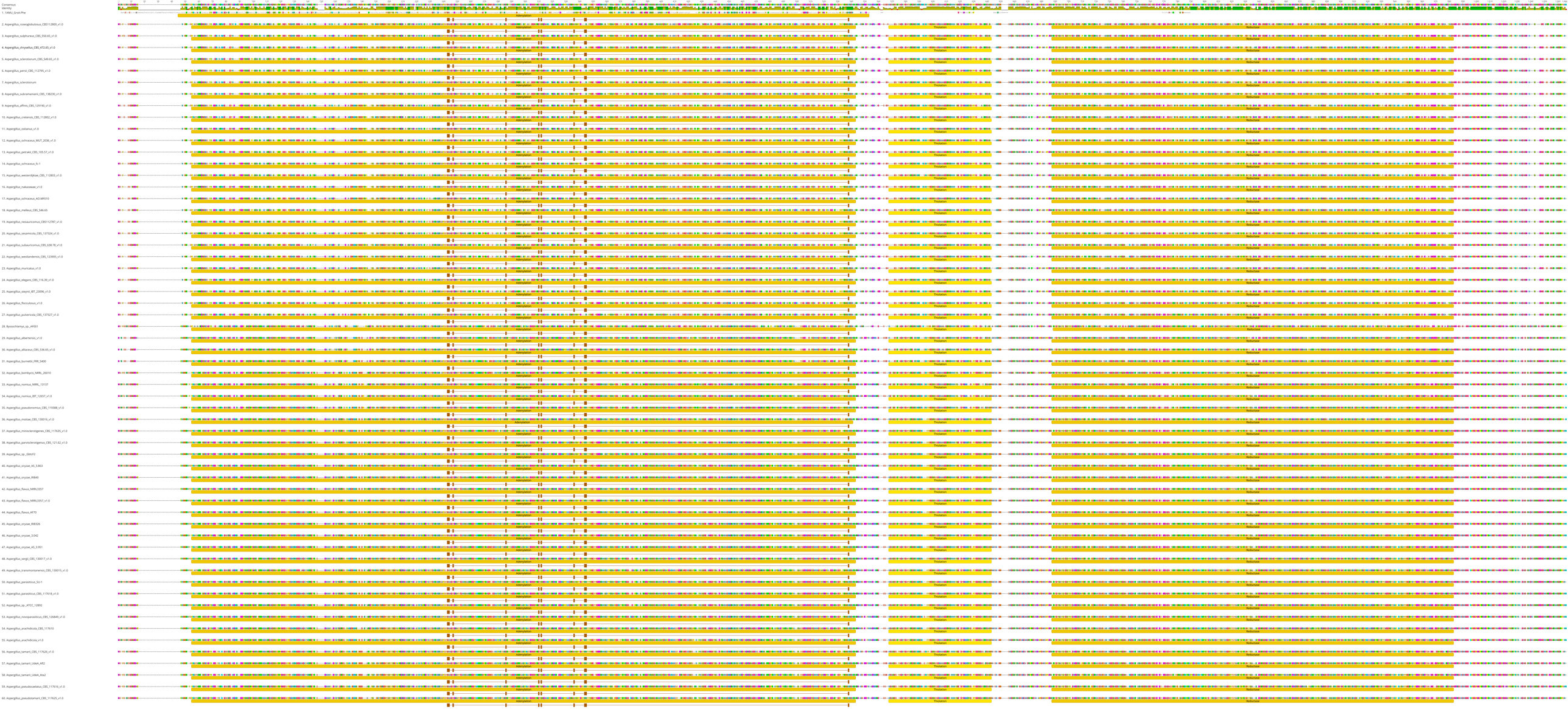

Supplement: Supplementary file 3 [file DataSheet_3.pdf]
